# A prognostic PET radiomic model for risk stratification in non-small cell lung cancer: integrating radiogenomics and clinical features to predict survival and uncover tumor biology insights

**DOI:** 10.1007/s00432-025-06232-8

**Published:** 2025-06-03

**Authors:** Parisa Taheri, Aaron Golden

**Affiliations:** 1 Physics, School of Natural Sciences, College of Science and Engineering, University of Galway, Galway, Ireland; 2 School of Mathematical and Statistical Sciences, College of Science and Engineering, University of Galway, Galway, Ireland; 3 Department of Medical Physics & Clinical Engineering, University Hospital Galway, Galway, Ireland

**Keywords:** ^18^F-FDG PET, NSCLC, Survival analysis, Radiomics, Radiogenomics

## Abstract

**Purpose:**

To develop a survival risk score using ^18^F-FDG PET radiomic features for non-small cell lung cancer (NSCLC) patients and to evaluate its biological basis as a prognostic radiomic signature through radiogenomic analyses.

**Methods:**

We utilized several NSCLC cohort datasets from the Cancer Imaging Archive (TCIA) for radiomic analysis, where transcriptomics data were available through the Cancer Genome Atlas (TCGA). A total of 945 radiomic features were extracted from the segmented tumors. A survival-based radiomic model was developed, from which a radiomic risk score was calculated. Radiogenomic analyses were then performed to explore correlations between radiomic risk cohorts and tumor transcriptomics, oncogenic pathways, and genetic mutations. We also constructed a nomogram by combining clinical and radiomic risk factors.

**Results:**

The PET-radiomic model significantly predicted the 5-year survival rate of patients, with AUCs of 0.78, 0.71, and 0.73 in the training, validation, and testing cohorts, respectively. Integration of clinical features and the radiomic risk score in a nomogram demonstrated enhanced efficacy, achieving AUCs greater than 0.85. Radiogenomic analysis revealed that while the low-risk group indicated anti-tumor immunity, the high-risk group exhibited transcriptomic characteristics associated with enhanced tumor aggressiveness, with consistent correlations between risk group membership, oncogenic pathways, immune cell types, and critical gene alterations.

**Conclusion:**

PET-radiomic features successfully delineated high- and low-risk NSCLC patient groups. Supporting radiogenomic analysis identified tumor-promoting characteristics and immune-suppressing activity in the high-risk group, which is consistent with these patients’ prognoses.

**Supplementary Information:**

The online version contains supplementary material available at 10.1007/s00432-025-06232-8.

## Introduction

Lung cancer is one of the most significant causes of cancer deaths worldwide, with non-small cell lung cancer (NSCLC) accounting for 85% of all cases (Cersosimo 2002). Despite significant advancements in diagnostic and therapeutic approaches, several challenges remain. The early diagnosis rate is still low, and diagnoses often occur after severe symptoms appear, which may lead to poor clinical outcomes. Long-term survival rates have also not seen substantial improvement, and the overall five-year survival rate for advanced-stage lung cancer patients is less than 2% (Chen et al. 2023).

In addition to these challenges, NSCLC is a heterogeneous disease consisting of diverse cell populations with distinct growth patterns and invasion tendencies. It impacts disease characteristics and makes it challenging to differentiate aggressive lung cancers from slower-growing tumors that may be curable by surgery (Hu et al. 2023). Traditionally, NSCLC risk assessment relies on clinical staging and biopsy-derived information. Although biopsy is the gold standard, it has limitations such as its invasive nature, being unrepeatable or impossible for inaccessible lung nodules, and providing only local information (Tang et al. 2023). Treatment outcomes may also vary in patients with the same stage (Arimura et al. 2019). Therefore, complementary systems are needed to provide additional information supporting personalized medicine (Qian et al. 2024).

Positron Emission Tomography (PET) is recommended by the National Comprehensive Cancer Network (NCCN) for NSCLC staging (Riely et al. 2024). As a non-invasive technique, PET provides metabolic insights based on differences in glucose metabolism across tissues, playing a crucial role in cancer management—both pre-treatment (staging, treatment planning) and post-treatment (response assessment and follow-up) (Sheikhbahaei et al. 2017).

Currently, PET images are assessed by the radiologist qualitatively or using semiquantitative features (SQFs), such as the maximum standardized uptake value (SUV_max_), which measure FDG uptake within a region of interest (ROI) (Ziai et al. 2016). However, the prognostic value of SUV_max_ remains uncertain. While some studies suggest that preoperative SUV_max_ is a significant prognostic factor in early-stage NSCLC, others report no significant association with survival in advanced resectable cases (Kaseda 2020). These discrepancies may be due to the limitations of SQFs in capturing spatial relationships among voxels. In contrast, PET radiomic features (RFs) complement standard macroscopic metrics (e.g., volume, SUVs) by quantifying intricate spatial patterns within tumors (Cook and Goh 2020).

Radiomics involves extracting quantitative image biomarkers from medical images using high-throughput algorithms. The central hypothesis behind radiomics is that RFs provide additional relevant information that is not visible to the naked eye but reflects underlying pathophysiological characteristics of the imaged volume (Lambin et al. 2012). The potential of radiomics, combined with advances in mapping human genes and mutations (e.g., from The Cancer Genome Atlas (TCGA) (Tomczak et al. 2015), and the availability of imaging resources (e.g., from The Cancer Imaging Archive (TCIA) (Clark et al. 2013), supports the use of radiogenomics to identify likely functional associations (Li and Zhou 2022). This methodological approach offers several advantages, including its non-invasive nature and ability to facilitate real-time treatment monitoring (Kaseda 2020).

Prognostic evaluation in patients is one of the earliest applications of radiomics in lung cancer (Chen et al. 2023). Several studies have utilized ^18^F-FDG PET texture features to predict disease-free and overall survival following surgical resection or chemoradiotherapy, identifying these features as potential biomarkers for improving prognosis and risk stratification (Cook et al. 2013; Ohri et al. 2016; Ahn et al. 2019; Dissaux et al. 2020). While radiomics has been correlated with multiple clinical outcomes, its biological significance remains unclear (Tomaszewski and Gillies 2021). Most studies primarily using Computed Tomography (CT) and Magnetic Resonance Imaging (MRI) have explored the likely underlying biology of structurally derived radiomic based prognostic predictions through radiogenomic analyses (Qian et al. 2024; Tomaszewski and Gillies 2021). However, given the unique potential of ^18^F-FDG PET to capture features directly associated with the molecular traits of tumors, understanding the genomic basis of PET prognostic models enables a more biologically informed basis to such risk stratification and so has the potential of providing both more robust assessments of tumor prognosis and also informed treatment decisions (Sheikhbahaei et al. 2017; Tomaszewski and Gillies 2021).

In the present study, we aimed to evaluate the prognostic value of a PET-radiomic survival model in NSCLC patients who underwent surgical treatment for which pre-treatment PET scan data was available and archived as part of TCIA. A substantial number of these patients enrolled in the TCGA consortium, which allowed us to explore multiple genomic characteristics, including oncogenic pathways, immune cell populations in the tumor microenvironment (TME), and gene mutations associated with the PET radiomic signature.

## Methods

### Datasets

Multiple datasets from TCIA/TCGA were utilized for our analysis. Data_1, the NSCLC-Radiogenomics dataset (Bakr et al. 2018), includes subjects with NSCLC who underwent preoperative PET/CT performed prior to tumor resection, with PET images scanned using a similar protocol. Data_2 comprises TCGA-LUAD (Albertina et al. 2016), TCGA-LUSC (Kirk et al. 2016), CPTAC-LUAD (National Cancer Institute Clinical Proteomic Tumor Analysis Consortium (CPTAC), 2018a), and CPTAC-LSCC (National Cancer Institute Clinical Proteomic Tumor Analysis Consortium (CPTACNational Cancer Institute Clinical Proteomic Tumor Analysis Consortium (CPTAC) 2018b), with radiology imaging collected from standard-of-care imaging performed immediately before pathological diagnosis. The clinical characteristics of patients in the cohorts are detailed in Supplementary Table S1.

### Study design

The PET radiomic (RAD) model was constructed using Data_1, which was chosen for model development due to its larger cohort size (*n* = 127). The subjects were randomly shuffled and partitioned into training and validation cohorts (70:30) for subsequent radiomic analysis. The resultant RAD model was evaluated in Data_2 (*n* = 43). We conducted a radiogenomic analysis in Data_2, which contains genomic data as part of TCGA. Only PET images were used to focus on the prognostic value of metabolic features, consistent with our study’s hypothesis. Subjects lacking key clinical or survival data, or with technical issues affecting image quality or processing, were excluded from the analysis (Supplementary Fig. S1).

### Radiomic model development

The primary tumor was delineated using *PET-IndiC (*Beichel et al. 2016*)*, a semiautomatic method that can be implemented as an extension of 3D Slicer. Nine PET SQFs were calculated during the segmentation process (Supplementary Table S2). A total of 945 RFs were then extracted from the segmented tumor by *PyRadiomics* (van Griethuysen et al. 2017) (Supplementary Table S3). Images were normalized and then resampled to isotropic voxels with 3 mm sides. A fixed bin width of 0.2 SUV_bw_ units was used to discretize voxel values.

During preprocessing, computed RFs underwent Z-score normalization. We assessed batch effects across datasets using principal component analysis (PCA). Highly correlated RFs (correlation coefficient > 0.80) were excluded through Pearson correlation assessment. Stable RFs with re-segmentation were selected based on concordance correlation coefficient (CCC > 0.85).

Univariate Cox analysis was applied to identify significant RFs. The most significant features were selected through the least absolute shrinkage and selection operator (LASSO) algorithm to limit redundancy, with the optimal lambda obtained through 3-fold cross-validation based on the maximum concordance index (CI). A robust multivariate model was constructed using multivariate Cox proportional hazards regression, with the best model chosen through bidirectional stepwise feature selection using the Akaike Information Criterion (AIC).

A radiomic (RAD) score for each patient was calculated by summing the product of each feature value and its corresponding weight assigned in the model. Patients were categorized into high-risk and low-risk groups using median and quantile values from the training data. Clinical and semiquantitative (SQ) models were built independently using the same methodology. A nomogram combining the RAD score and other risk factors was also constructed. We additionally performed hierarchical clustering analysis on the radiomic features that were selected following univariate Cox analysis to identify high- and low-risk clusters.

### Radiogenomic analyses

Genomic data (gene expression and copy number variation (CNV)) for Data_2 subjects were obtained from the TCGA Genomic Data Commons (https://portal.gdc.cancer.gov/). Gene expression data were downloaded as raw counts and TPM-normalized values; raw counts were used for differential expression analysis, and TPM-normalized data for radiogenomic comparisons. Gene set enrichment analysis (GSEA) was performed to assess pathways enriched in PET-radiomics risk cohorts. We converted gene expressions to pathway enrichment scores per sample using gene set variation analysis (GSVA). Pathway gene sets were sourced from the Molecular Signatures Database (MSigDB) (https://www.gsea-msigdb.org/gsea/msigdb).

The association between patient risk scores and genomic CNV scores was assessed. For each patient, amplification and deletion scores were calculated as the number of genes with positive and negative CNVs (threshold of ± 0.15), respectively; the overall CNV score was the sum of these values.

### Statistical analysis

Statistical and genomics analyses were conducted using *R* (v4.3.3) and *Python* (v3.9.15). Overall survival (OS), defined as time from diagnosis to death or the last follow-up, served as the clinical endpoint. We employed univariate Cox regression, Coxnet, and multivariate stepwise Cox analysis to determine independent predictors in NSCLC patients. Model performance was assessed using the area under the curve (AUC) of receiver operating characteristic (ROC) curves and Kaplan-Meier (KM) plots. We used the Wilcoxon signed-rank test and two-tailed t-test to compare quantitative variables, and Fisher’s exact test to explore correlations between ordinal variables. Statistical significance was set as *p* ≤ 0.05, with multiple testing correction via the Benjamini-Hochberg method (p-adjust ≤ 0.05).

## Results

### Radiomic model development

**Fig. 1:**
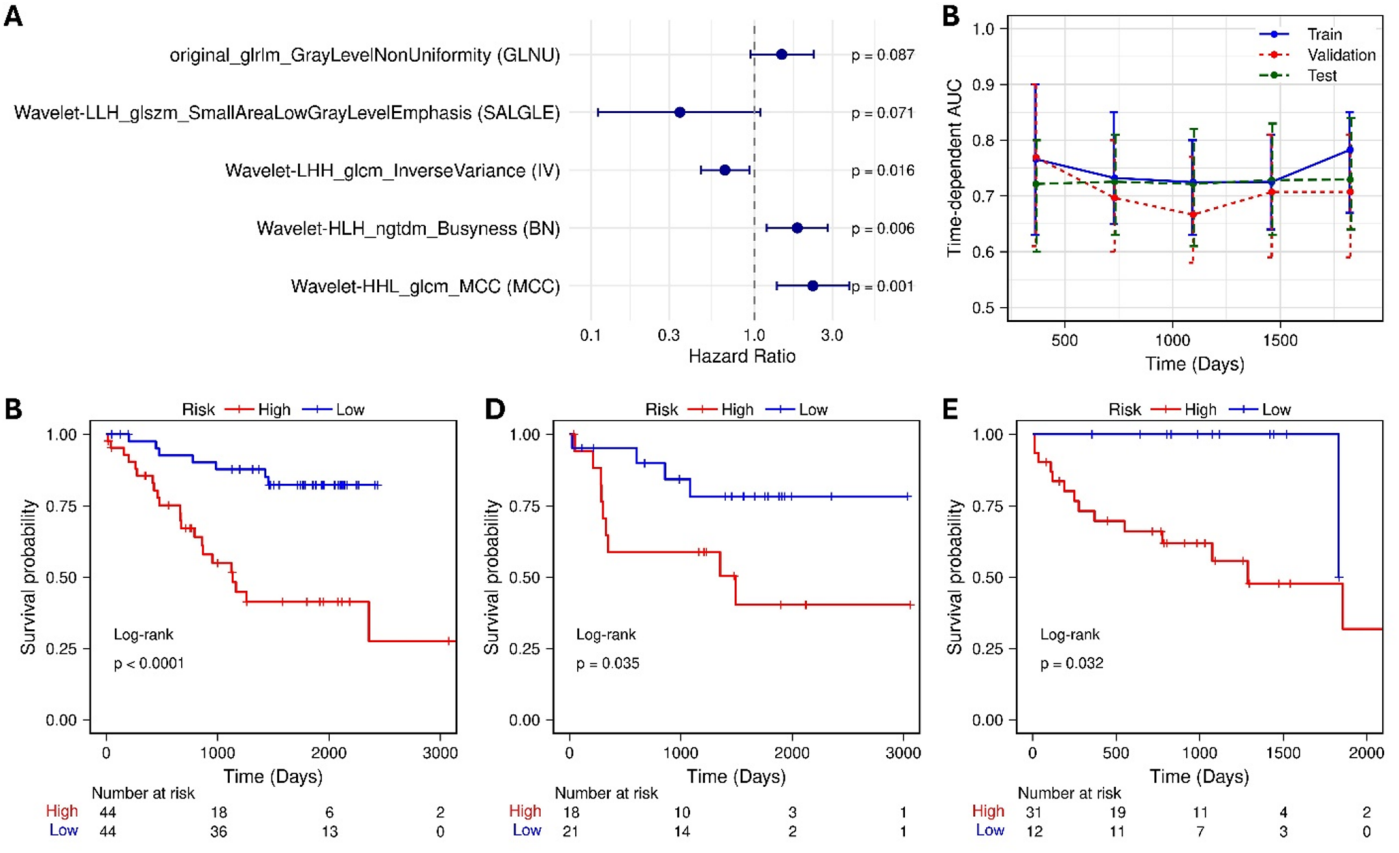
(**A**) Forest plot displaying the hazard ratios (HRs) and 95% confidence intervals (CIs) for radiomic features included in the RAD model. (**B**) Time-dependent AUC values of the radiomic model across different cohorts. Kaplan-Meier (KM) analysis comparing survival probabilities between high and low RAD score groups in the (**C**) training, (**D**) validation, and (**E**) test cohorts

Since Data_1 and Data_2 were obtained from different institutions, we assessed potential batch effects across RFs using PCA. The PCA results indicated no clear separation between the Data_1 and Data_2 cohorts in component space (Supplementary Fig. S2). Following stability assessment, 898 RFs were retained for further analysis, of which 445 were significantly associated with OS in univariate Cox analysis. After correlation filtering, 31 non-redundant features remained. Due to their strong correlation with the outcome, we employed the LASSO algorithm (optimal lambda: 0.07; Supplementary Fig. S3) and bidirectional stepwise feature selection to develop a radiomic model incorporating the five remaining RFs (Fig. 1A; Supplementary Table S4). Three-fold cross-validation was used to select the optimal lambda during LASSO regularization, balancing model stability and sample size. Post hoc comparisons with 5- and 10-fold cross-validation confirmed the robustness of the selected features, with 10-fold identifying a few additional ones. Variance inflation factor (VIF) analysis on the final RFs showed low multicollinearity (all VIFs < 2; mean ~ 1.4). The RAD score was calculated using the following formula:

$$\begin{aligned}\text{RAD\:score}&=\left(\text{GLNU}\right)\times\:\left(0.39\right)\\&\quad+(\text{SALGLE)\:}\times\:\left(-1.05\right)\\&\quad+\text{\:(IV)}\:\times\:\left(-0.42\right)\\&\quad+(\text{BN\:})\times\:\left(0.61\right)+\left(\text{MC}\text{C}\right)\times\left(0.83\right)\end{aligned}$$

As shown in Fig. 1B, the RAD model achieved an average AUC above 0.70, with an AUC of 0.73 in the testing cohort, and demonstrated significant risk stratification (Figs. 1C-E; log-rank test; *p* < 0.05). Unsupervised clustering of 445 radiomic features (identified via univariate Cox analysis) revealed two optimal clusters based on silhouette analysis. These clusters showed significantly different survival outcomes in KM analysis (*p* = 0.008) and were clearly separated in PCA space. Additionally, the low- and high-risk clusters were significantly associated with oncologic pathways and the RAD-score from the multivariate Cox model (Fisher’s exact test: *p* = 0.00001) (Supplementary Fig. S4).

### Clinical and semiquantitative PET features

To compare and integrate radiomics with other data, we also assessed clinical risk factors (e.g., age, gender, histology) and PET SQFs (e.g., SUV_max_, SUV_mean_, MTV) for the same patient cohorts. A clinical model was constructed using the same survival analysis formalism previously described (Supplementary Table S5; Supplementary Fig. S5).

We also independently evaluated PET SQFs. Due to their high correlation with one another (Supplementary Fig. S6), only SUV_max_ and MTV were further analyzed. SUV_max_ was then selected by the Cox stepwise method (Supplementary Fig. S7), and the semiquantitative (SQ) score for each subject was computed using the estimated regression coefficient, given by the formula:$\:\text{SQ\:score}=\:\:(\text{SUVmax)\:}\times\:\left(0.20\right)$

**Fig. 2:**
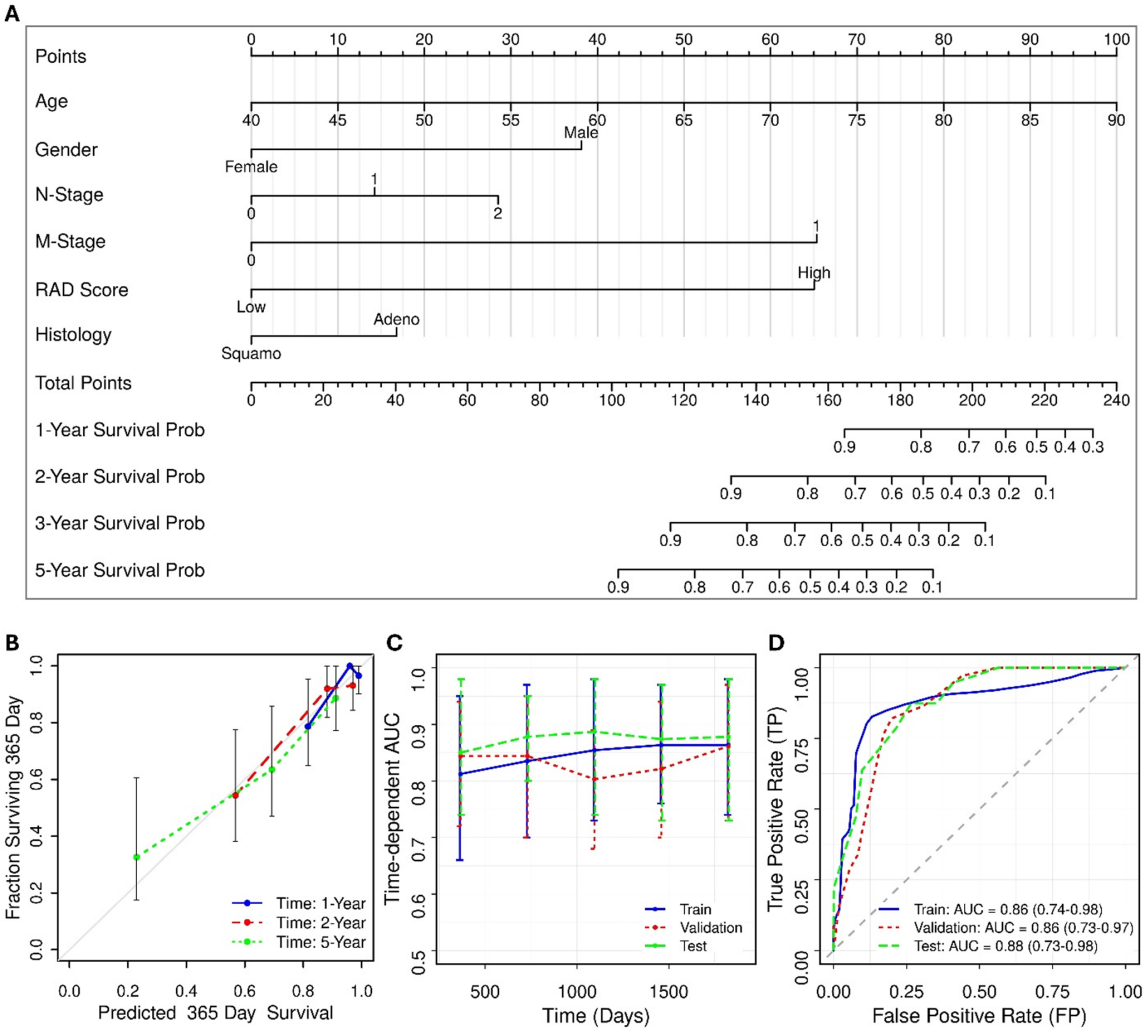
(**A**) Nomogram combining the RAD score with significant clinical risk factors. (**B**) Calibration curves of the nomogram. (**C**) Time-dependent AUC values of the nomogram. (**D**) Time dependent ROC curves for the 5-year survival rate estimated by the nomogram

**Fig. 3:**
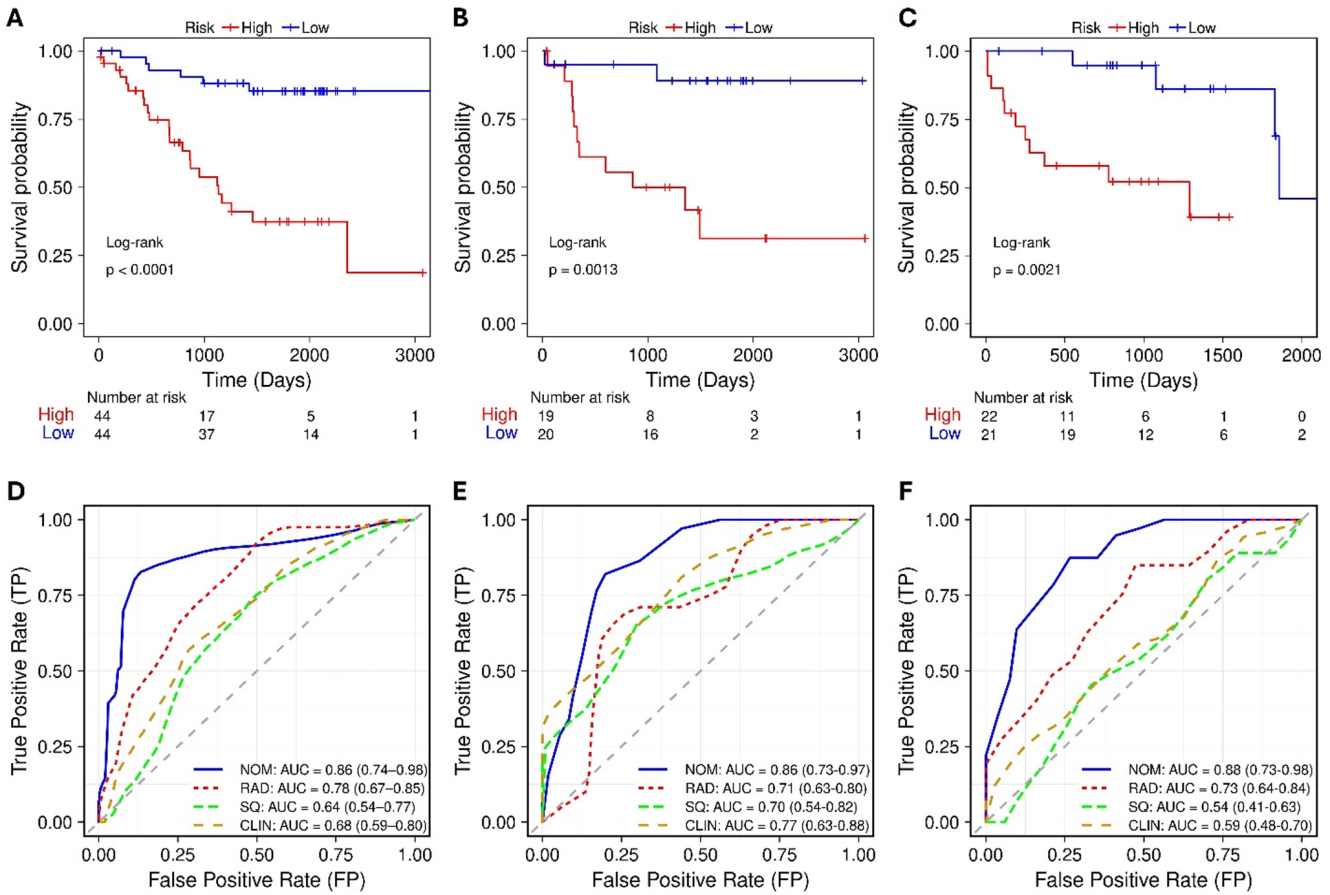
KM plots of nomogram (top) and ROC curves (bottom) of different clinical (CLIN), radiomics (RAD), semiquantitative (SQ), and nomogram (NOM) models in the (**A**,** D**) training, (**B**,** E**) validation, and (**C**,** F**) test datasets

To further refine our predictions, we integrated our RAD score and clinical features into a nomogram (Fig. 2; Supplementary Table S6). The SQ score (SUV_max_) was excluded from this analysis due to its high correlation with four of the five RFs including GLNU, SALGLE, IV, and MCC (*p* < 0.01), as well as with the RAD score (*r* = 0.60; *p* < 0.0001) (Supplementary Fig. S8). The nomogram consistently estimates survival and significantly enhances predictive performance compared to other models (Fig. 3).

### Radiogenomic analyses

**Fig. 4:**
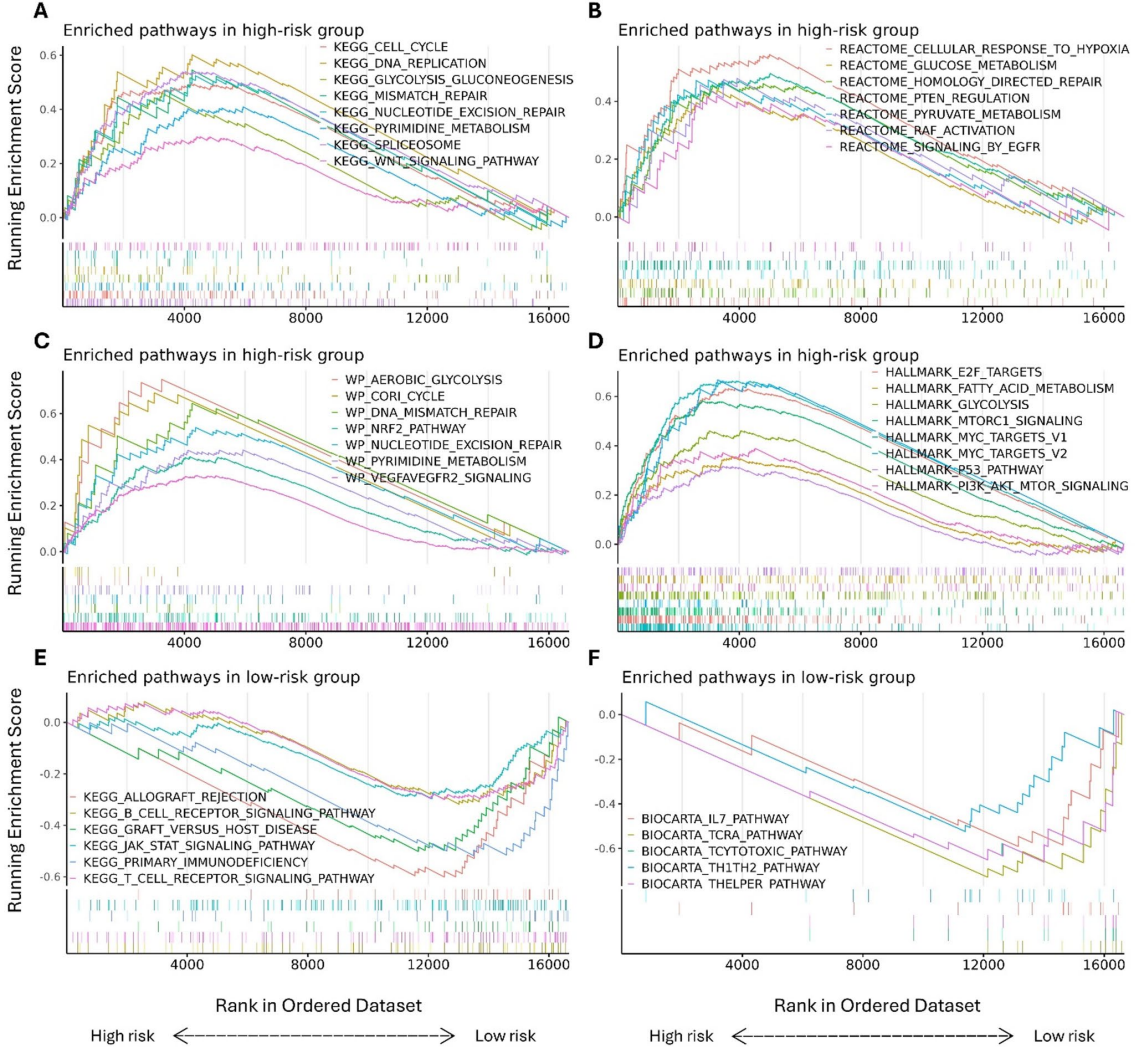
Gene set enrichment analysis (GSEA), showcasing top significantly enriched biological pathways derived from various gene set collections (**A-D**) in high-risk group patients and (**E**,** F**) in low-risk group patients. Enrichment results are ranked based on normalized enrichment score (NES), and only pathways with FDR < 0.05 are shown

GSEA analysis of differential expressed genes (p-adjust < 0.05; logFC > 1.5) across the risk groups/clusters resulted in molecular pathways associated with risk stratification as illustrated in Fig. 4. Patients with high PET-RAD scores exhibited significant enrichment of pathways relayed to the cell cycle, metabolism, glycolysis, and nucleotide excision repair (NER) (p-adjust < 0.05). Conversely, low-risk patients showed enrichment in immunity-related pathways and tumor microenvironment interactions, such as the T-cell/B-cell receptor pathway.

**Fig. 5:**
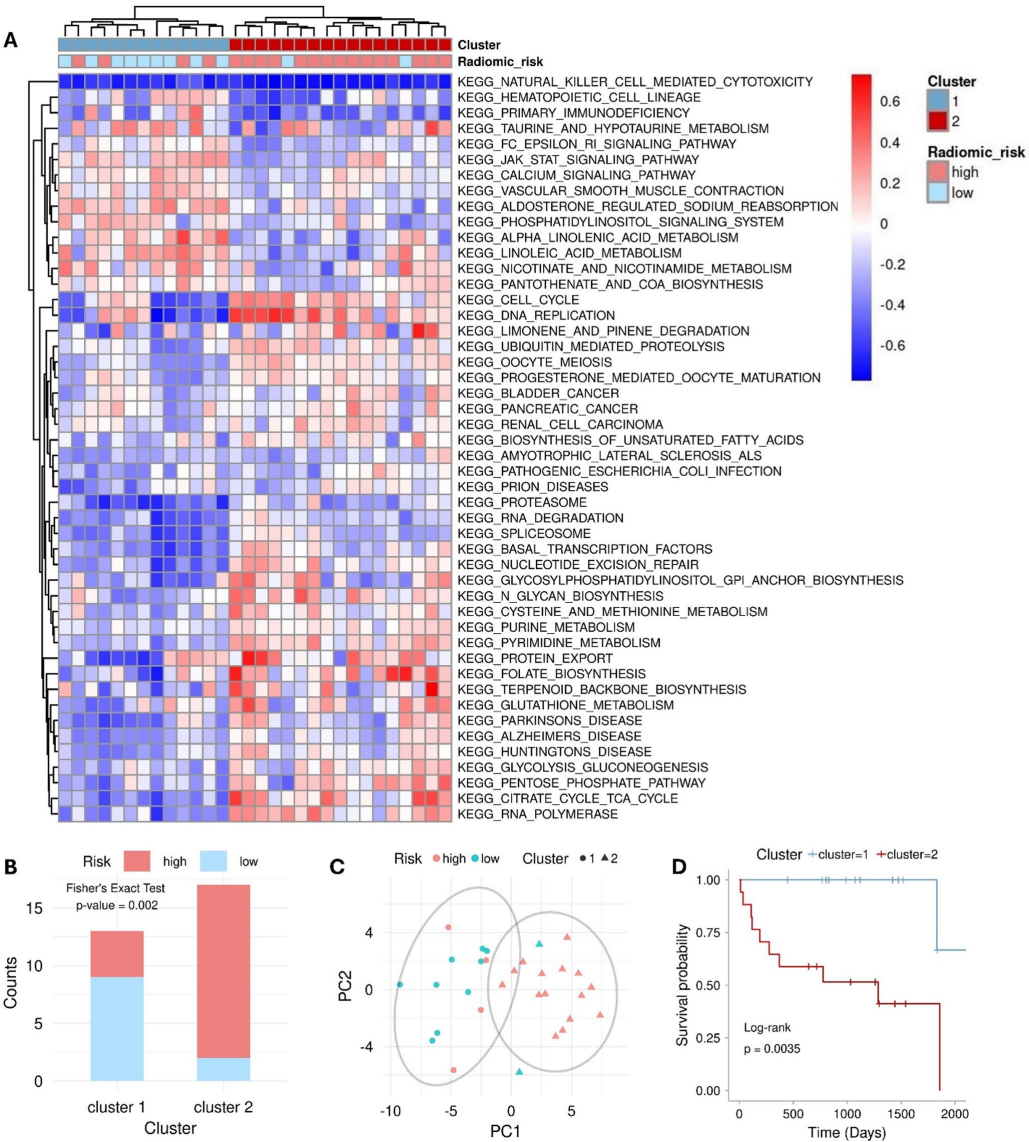
Gene set variation analysis (GSVA). (**A**) Clustering analysis of the KEGG functional enrichment score for each sample, acquired through GSVA. (**B**) Correlation assessment between RAD scores and clusters. (**C**) PCA analysis of GSVA scores. (**D**) K-M analysis of clusters

Our inferred cell infiltration analysis revealed that immune cell types, such as CD8 + T cells, CD4 + memory T cells, and B cells, were more enriched in the low-risk cohort (*p* < 0.05), suggesting immune surveillance functions. The immune score was also higher in the low-risk cohort (*p* = 0.057), explaining the more favorable prognosis in this group (Supplementary Fig. S9). Figure 5 shows distinct KEGG pathways classified patients into two specific clusters, with significant associations between these clusters, risk subgroups and survival outcomes (Fisher’s exact test: *p* = 0.002; KM analysis: *p* = 0.004).

**Fig. 6:**
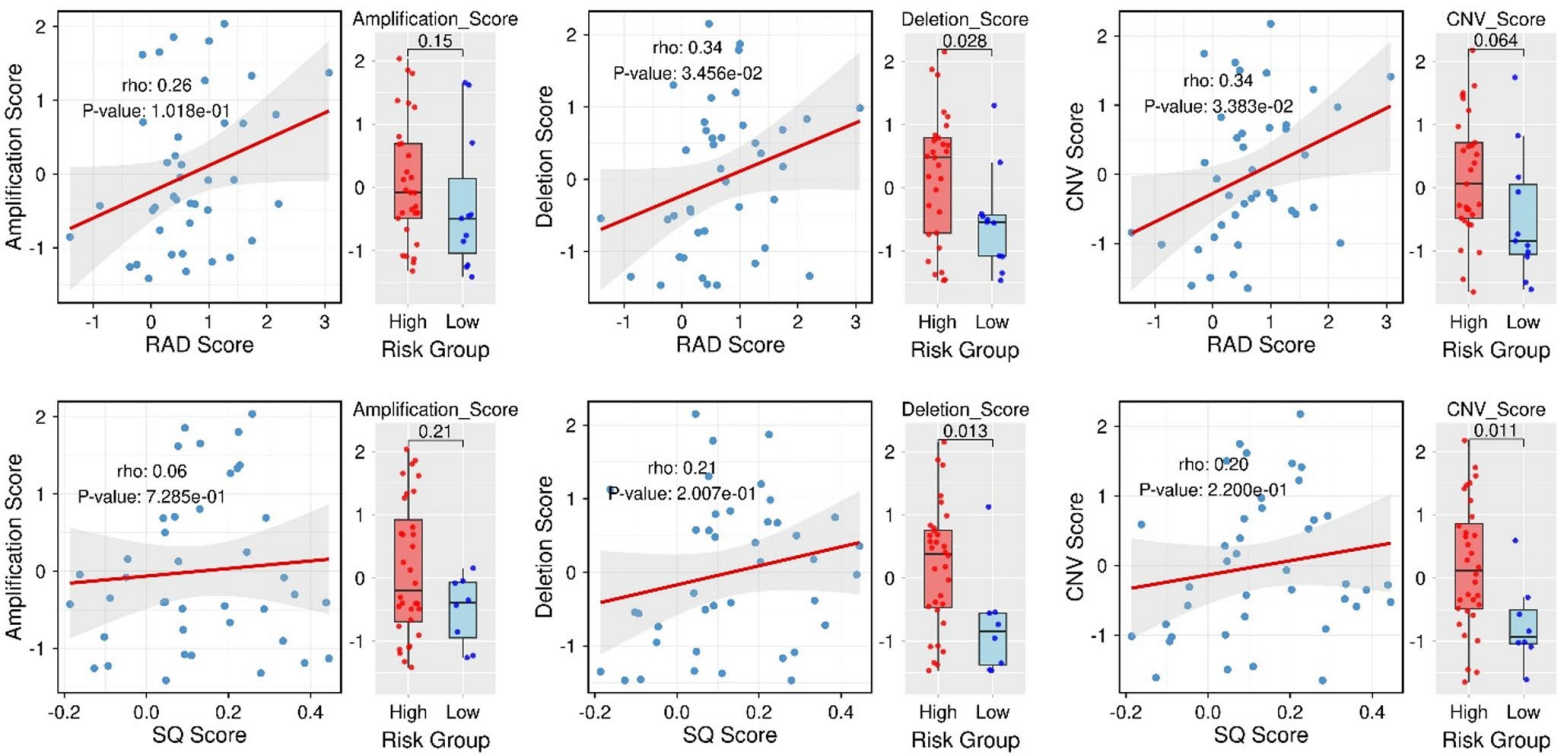
Top: CNV scores vs. RAD scores, including comparisons between the RAD-risk groups. Bottom: CNV scores vs. SQ scores, including comparisons between the SQ-risk groups

Further CNV assessment revealed significant correlations between the RAD score and both the deletion score (*r* = 0.34, *p* = 0.03) and the overall CNV score (*r* = 0.34, *p* = 0.03), but no significant association with the amplification score (Fig. 6). Although SQ score values were not correlated with CNV scores, they showed differences across the high and low SQ risk groups for the deletion score and the overall CNV score (Wilcoxon test; *p* = 0.01). Multiple genes from key biological pathways were significantly associated with both RAD and SQ scores, with several of these associations accompanied by frequent copy number gains and losses (Supplementary Tables S7–S10). Additionally, many of these genes showed correlations with survival outcomes (Supplementary Fig. S10).

## Discussion

PET/CT RFs have been assessed in previous studies for prognosis evaluation (Cook et al. 2013; Ohri et al. 2016; Ahn et al. 2019; Dissaux et al. 2020). However, most of these studies have evaluated only a limited number of RFs. Additionally, while RFs have been incorporated into such prognostic models, validation beyond an independent test cohort, particularly with biological aspects, remains limited (Tomaszewski and Gillies 2021), especially in PET-radiomics (Qian et al. 2024), where the focus has been on the identification of RFs indicative of treatment response and/or genotype status. Several studies have explored the biological significance of prognostic radiomic signatures derived using survival analysis or other supervised methods, primarily focusing on CT radiomics (Aerts et al. 2014; Lee et al. 2018; Xie et al. 2020). Hannequin et al. (Hannequin et al. 2022) developed a PET/CT prognostic signature focused on tumor PD-L1 expression, but neither PET nor CT RFs in their study significantly distinguished PD-L1 status. Our work represents, to our knowledge, the first such study to rigorously explore the underlying molecular biology associated with the PET radiomic signature linked to prognostic risk.

**Fig. 7:**
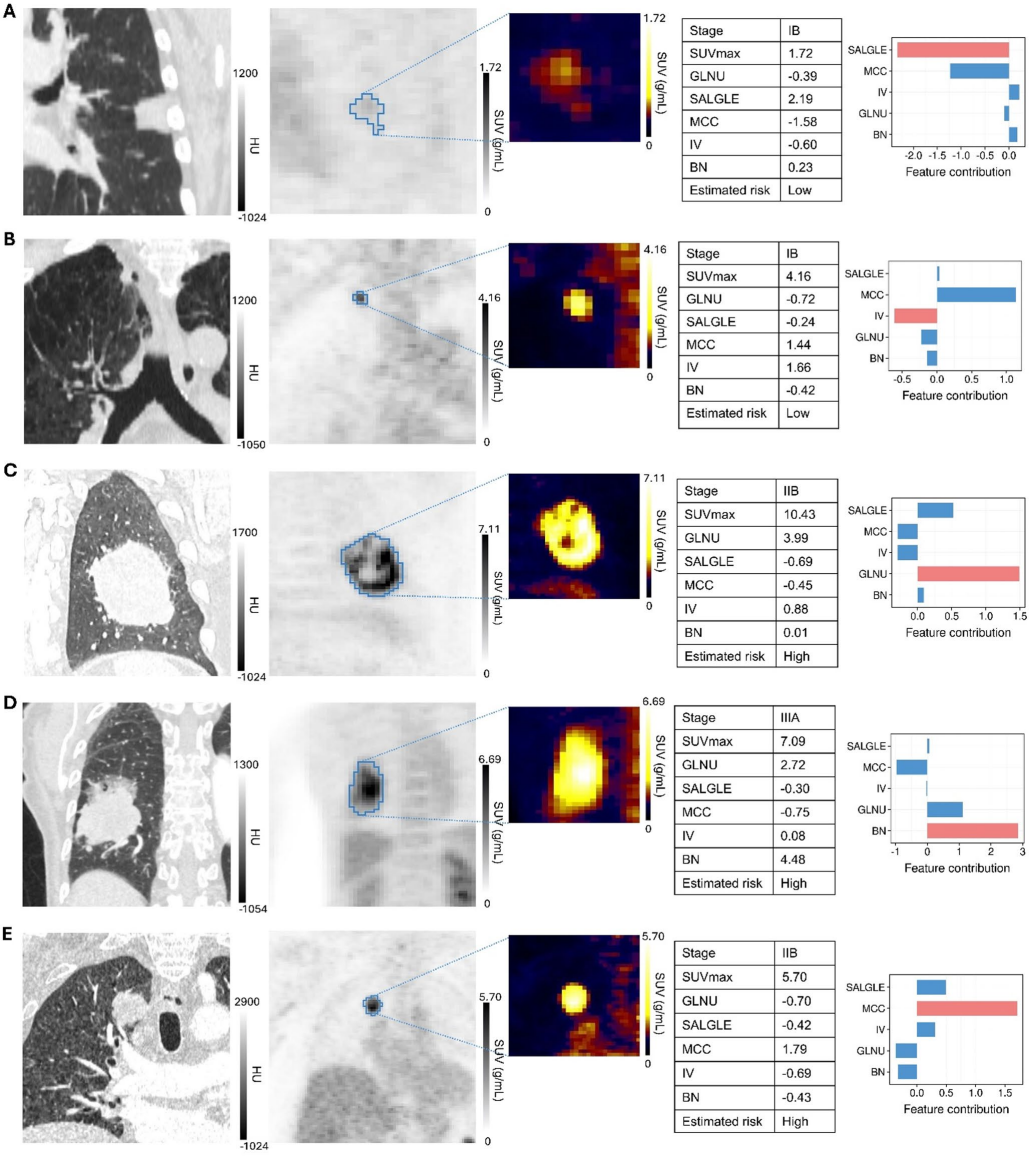
Instances of radiomic feature values, importance, and segmented ROIs. (**A**) SALGLE, linked to better prognosis. (**B**) IV, with higher values in low-risk patients. (**C-E**) Higher values of GLNU, BN, and MCC, respectively, in tumors with poorer prognoses. Supplementary Table S11 contains more details on the range of RFs and risk scores

Our NSCLC PET-radiomic model had as its basis five specific RFs that were selected on account of their consistent robust correlation with patient prognosis. To assess the stability of this selection, we repeated feature selection using bootstrapping within the training data, which yielded a substantially overlapping feature set, supporting the robustness of our pipline. In Fig. 7, we present separate examples of high and low risk cases with associated RF contributions to each patient’s risk assessment. Better prognosis was shown to be positively associated with the RF *SmallAreaLowGrayLevelEmpasis* (SALGLE) (Fig. 7A), which is linked to diminished metabolic activity due to lower ^18^F-FDG-uptake in the tumor region (Supplementary Fig. S8B), and the RF *wavelet_LHH_glcm_InverseVariance* (IV), which is associated with tissue homogeneity (Fig. 7B). Tumors with worse prognoses exhibited higher values of the RFs *original_glrlm_GrayLevelNonUniformity* (GLNU) and *wavelet-HLH_ngtdm_Busyness* (BN) (Figs. 7C-D). Collectively, these features capture the more heterogeneous tumor tissue texture and are consistent with the consensus that highly differentiated tumors possess a diversity of regional proliferation, hypoxia, angiogenesis, and necrosis tendencies, and form the basis for their innate aggressiveness (Cook et al. 2013). Tumors with poor prognosis were noted as having consistently high busyness texture features derived from the NGTDM class in the study by Ahn et al. (Ahn et al. 2019), consistent with our findings. In several aggressive cases, high values of the RF *wavelet-HHL_glcm_MCC* (MCC) were identified (Fig. 7E), typically accompanied by a higher SUV_max_ indicative of a more metabolically active region (Supplementary Fig. S8E).

Leveraging each patient’s comprehensive genomic information against their risk status enabled us to substantiate the likely genotype conditions associated with the prognoses derived from our radiomic analysis. Notably, we observed a consistent association between signatures of an active immune environment and membership in the low-risk cohort. Dysregulated pathways, such as the cell cycle, proliferation, angiogenesis, and NER pathway dominated in high-risk patients. In contrast however, the low-risk group consistently exhibited activation in tumor suppression pathways and primary immunodeficiency.

These pathway differences demonstrate the biological relevance of our radiomic risk models. In the high-risk group/cluster, enrichment of cell cycle, glycolysis/gluconeogenesis, and DNA replication pathways suggests increased proliferation and metabolic reprogramming—hallmarks of aggressive tumors and poor prognosis in early-stage resected NSCLC (Kaira and Yamamoto 2010; Šutić et al. 2021).

Conversely, the activation of immune-related pathways in the low-risk group—including T/B cell receptor, T helper cell, Cytotoxic T Cell, and allograft rejection pathways—suggests an immunologically “hot” tumor microenvironment, which is associated with better therapeutic response and favorable survival (Liberini et al. 2021). Supporting this, transcriptomic-derived immune cell infiltration analysis indicated enhanced presence of immune effectors such as CD8+/CD4 + T cells, in the TME of the low-risk cohort, reinforcing improved immune surveillance.

Furthermore, the genomic landscape of the high-risk group revealed frequent copy number gains in oncogenes such as *EGFR*, *SLC2A1*, and *HIF-1 A* alongside losses in key tumor suppressors like *PTEN*, *TP53*, *BRCA1*, *ERCC1*, and *CDKN2A*. These alterations are consistent with activation of proliferative and survival pathways, including PI3K/AKT signaling and NER, which are known contributors to treatment resistance and poor prognosis in resected NSCLC (Rose-James and Tt 2012). Elevated ^18^F-FDG uptake on PET reflects this altered metabolic activity (Warburg effect), serving as a potential prognostic factor. This uptake is mediated by the glucose transporter (Glut1; SLC2A1), which is believed to be regulated by hypoxia-inducible factor 1-alpha (HIF-1 A) (Kaira and Yamamoto 2010).

The convergence of these molecular alterations with RFs supports a biologically plausible link. Tumors with increased metabolic activity, proliferation, and genomic instability often exhibit greater PET signal intensity and heterogeneity. RFs associated with the low-risk group—such as lower gray-level values (SALGLE) and higher textural uniformity (IV)—may reflect reduced glycolytic activity and enhanced immune cell infiltration within the TME, which aligns with prior studies indicating that more homogeneous textures are associated with a more active, less exhausted immune environment (Bouhamama et al. 2023; Zhang et al. 2023). These findings suggest that PET-derived RFs could serve as non-invasive biomarkers of tumor aggressiveness and immune infiltration in resected NSCLC, helping to identify patients who may benefit from intensified post-treatment surveillance or immunotherapy.

We acknowledge that correlation-based radiogenomic analyses are inherently inferential. Further validation using immunohistochemistry or proteomic profiling would be valuable to confirm the observed associations. Our study was also limited by the small patient sample available in the National Cancer Institute’s public database; however, this is partially offset by the comprehensive and well-controlled data types available for scrutiny. Additionally, PET’s low spatial resolution may impact segmentation accuracy and feature stability, particularly in small tumors. To address this, we applied image resampling, robust multi-segmentation-based feature stability assessment, and a semi-automated algorithm with high inter-/intra-operator agreement compared to manual segmentation. To further validate PET-radiomics as a noninvasive biomarker for risk assessment, we are investigating our model using real-world patient data from our institution. Identifying high-risk patients through such approaches could support clinical decision-making and selecting candidates for adjuvant or neoadjuvant therapies. Nonetheless, future studies are needed to determine whether such radiogenomic approach provides sufficient clinical benefit to be integrated into treatment planning.

## Conclusion

In this study, we developed a survival-based PET-radiomic signature, which effectively stratified NSCLC patients based on prognostic risk. The radiomic risk score was significantly correlated with genetic characteristics obtained through analyzing RNA-sequencing, copy number alteration, and gene mutation data, providing biological insight into why high-risk subjects had a worse prognosis than low-risk patients, in particular highlighting the active immune environment for the latter. Compared to previous work that focused mainly on CT and MRI, our results support the integration of PET radiomics into precision oncology workflows, which allows for a biologically informed, evidence-based risk estimator, improving treatment selection and personalized interventions for lung cancer patients.

## Electronic supplementary material

Below is the link to the electronic supplementary material.

Supplementary Material 1

## Data Availability

No datasets were generated or analysed during the current study.
