# Supplementary material for: A prognostic PET radiomic model for risk stratification in non-small cell lung cancer: integrating radiogenomics and clinical features to predict survival and uncover tumor biology insights
Source: J Cancer Res Clin Oncol. 2025 Jun 3;151(6):180. doi: 10.1007/s00432-025-06232-8 (PMC12130170; doi:10.1007/s00432-025-06232-8)
Supplement: Supplementary file 1 — Supplementary Material 1 [file 432_2025_6232_MOESM1_ESM.docx]

# Supplementary Data

## Supplementary Tables

**Supplementary Table S1.** Patient clinical characteristics in training, internal validation and external validation datasets

| Characteristic | Data_1 | | | | Data_2 | |
| --- | --- | --- | --- | --- | --- | --- |
|  | **Train (n=88)** | | **Validation (n=39)** | | **Test (n=43)** | |
|  | **n (%), Range (Mean)** | **p-value ^*^** | **n (%), Range (Mean)** | **p-value ^*^** | **n (%), Range (Mean)** | **p-value ^*^** |
| Age | 43-86; (68) | 0.26 | 46-87; (69) | 0.07 | 45-83 (69) | 0.30 |
| Gender |  |  |  |  |  |  |
| Male | 62 (70%) | 0.32 | 29 (74%) | 0.16 | 19 (56%) | 0.51 |
| Female | 26 (30%) |  | 10 (26%) |  | 24 (44%) |  |
| Overall stage |  | 0.19 |  | 0.001 |  | 0.32 |
| I-II | 73 (83%) |  | 32 (82%) |  | 34 (79%) |  |
| III-IV | 15 (17%) |  | 7 (18%) |  | 9 (21%) |  |
| T Stage |  | 0.42 |  | 0.10 |  | 0.57 |
| 1 | 39 (44%) |  | 20 (51%) |  | 9 (21%) |  |
| 2 | 34 (39%) |  | 12 (31%) |  | 25 (58%) |  |
| 3 | 12 (14%) |  | 6 (15%) |  | 7 (16%) |  |
| 4 | 3 (3%) |  | 1 (3%) |  | 2 (5%) |  |
| N Stage |  | 0.03 |  | 0.007 |  | 0.26 |
| 0 | 70 (80%) |  | 33 (85%) |  | 30 (70%) |  |
| 1 | 10 (11%) |  | 5 (13%) |  | 8 (18%) |  |
| 2 | 8 (9%) |  | 1 (3%) |  | 5 (12%) |  |
| M Stage |  | 0.14 |  | 0.02 |  | 0.04 |
| 0 | 84 (95%) |  | 38 (97%) |  | 42 (98%) |  |
| 1 | 4 (5%) |  | 1 (3%) |  | 1 (2%) |  |
| Histology |  |  |  |  |  |  |
| Adenocarcinoma | 65 (74%) | 0.97 | 32 (82%) | 0.03 | 18 (42%) | 0.32 |
| Squamous cell carcinoma | 21 (24%) |  | 6 (15%) |  | 25 (58%) |  |
| Not specified | 2 (2%) |  | 1 (3%) |  | 0 (0%) |  |
| SUV_max_ | 0.84-35.66; (8.40) | 0.16 | 0.98-22.15; (6.56) | 0.001 | 1.67-27.89; (12.33) | 0.30 |
| Survival status |  |  |  |  |  |  |
| Dead | 29 (33%) |  | 13 (33%) |  | 16 37%) |  |
| Alive | 59 (67%) |  | 26 (67%) |  | 27 (63%) |  |
| Categorical variables are presented as n (%), and continuous variables as range (mean). | | | | | | |
| ^*^ The p-value indicates the significance of each feature in univariate Cox analysis. | | | | | | |

**Supplementary Table S2.** Conventional PET features calculated during ROI segmentation.

| Feature | Index | Units |
| --- | --- | --- |
| Mean uptake value | SUV_mean_ | SUV_bw_ |
| Minimum uptake value | SUV_min_ | SUV_bw_ |
| Maximum uptake value | SUV_max_ | SUV_bw_ |
| Peak value within ROI | SUV_peak_ | SUV_bw_ |
| Sum of segmented voxel volumes | Volume | ml |
| Total Lesion Glycolysis | TLG | SUV_bw_*ml |
| Standard Deviation of uptake values | SUV_SD_ | SUV_bw_ |
| Median uptake value | SUV_median_ | SUV_bw_ |
| Standardized added metabolic activity | SAM | SUV_bw_*ml |

**Supplementary Table S3.** Radiomic features based on shape, histogram, and texture.

| Feature group | Image Type ^*^ | Feature Class | Feature Name |
| --- | --- | --- | --- |
| Shape-based (2D and 3D) | Original | Shape | Elongation, Flatness, LeastAxisLength, MajorAxisLength, Maximum2DDiameterColumn, Maximum2DdiameterRow, Maximum2DdiameterSlice, Maximum3Ddiameter, MeshVolume, MinorAxisLength, Sphericity, SurfaceArea, SurfaceVolumeRatio, VoxelVolume |
| First-order statistics (intensity or histogram based) | \| Original \| \| --- \| \| Wavelet (High  and low pass  in x,y and  z directions) \| \| Log-sigma  (σ=1.0) \| | First-order | 10Percentile, 90Percentile, Energy, Entropy InterquartileRange, Kurtosis, Maximum, MeanAbsoluteDeviation, Mean, Median, Minimum, Range, RobustMeanAbsoluteDeviation, RootMeanSquared, Skewness, TotalEnergy, Uniformity, Variance |
| High-order (textural) | \| Original \| \| --- \| \| Wavelet (High  and low pass  in x,y and  z directions) \| \| Log-sigma  (σ=1.0) \| | Gray Level Co-occurrence Matrix  (GLCM) | Autocorrelation, ClusterProminence, ClusterShade, ClusterTendency, Contrast, Correlation, DifferenceAverage, DifferenceEntropy, DifferenceVariance, Id, Idm, Idmn, Idn, Imc1, Imc2, InverseVariance, JointAverage, JointEnergy, JointEntropy, MCC, MaximumProbability, SumAverage, SumEntropy  SumSquares |
|  |  | Gray Level Size Zone Matrix (GLSZM) | GrayLevelNonUniformity, GrayLevelNonUniformityNormalized, GrayLevelVariance, HighGrayLevelZoneEmphasis, LargeAreaEmphasis, LargeAreaHighGrayLevelEmphasis, LargeAreaLowGrayLevelEmphasis, LowGrayLevelZoneEmphasis, SizeZoneNonUniformity, SizeZoneNonUniformityNormalized, SmallAreaEmphasis |
|  |  | Gray Level Run Length Matrix (GLRLM) | GrayLevelNonUniformity, GrayLevelNonUniformityNormalized, GrayLevelVariance, HighGrayLevelRunEmphasis, LongRunEmphasis, LongRunHighGrayLevelEmphasis, LongRunLowGrayLevelEmphasis, LowGrayLevelRunEmphasis, RunEntropy, RunLengthNonUniformity, RunLengthNonUniformityNormalized, RunPercentage, RunVariance, ShortRunEmphasis, ShortRunHighGrayLevelEmphasis, ShortRunLowGrayLevelEmphasis |
|  |  | Neighbouring Gray Tone Difference Matrix (NGTDM) | Busyness, Coarseness, Complexity, Contrast, Strength |
|  |  | Gray Level Dependence Matrix (GLDM) | DependenceEntropy, DependenceNonUniformity, DependenceNonUniformityNormalized, DependenceVariance, GrayLevelNonUniformity  GrayLevelVariance, HighGrayLevelEmphasis, LargeDependenceEmphasis, LargeDependenceHighGrayLevelEmphasis, LargeDependenceLowGrayLevelEmphasis, LowGrayLevelEmphasis, SmallDependenceEmphasis, SmallDependenceHighGrayLevelEmphasis, SmallDependenceLowGrayLevelEmphasis |
| * Radiomic features were extracted from the original and filtered (wavelet and Laplacian-of-Gaussian (LoG)) images. Wavelet filters were applied as high/low band-pass in the x, y, and z directions. For LoG filtering, we used the sigma value of 1.0. | | | |

**Supplementary Table S4.** Multivariate Cox regression analysis of PET-radiomic features

| Feature | P-value | HR^*^ | 95%CI^†^ |
| --- | --- | --- | --- |
| original_glrlm_GrayLevelNonUniformity (GLNU) | 0.087 | 1.47 | 0.95-2.30 |
| Wavelet-LLH_glszm_SmallAreaLowGrayLevelEmphasis (SALGLE) | 0.071 | 0.35 | 0.11-1.09 |
| Wavelet-LHH_glcm_InverseVariance (IV) | 0.016 | 0.66 | 0.47-0.93 |
| Wavelet-HLH_ngtdm_Busyness (BN) | 0.006 | 1.83 | 1.19-2.82 |
| Wavelet-HHL_glcm_MCC (MCC) | 0.001 | 2.28 | 1.37-3.80 |
| * HR, hazard ratio |  |  |  |
| ^†^ CI, confidence interval |  |  |  |

**Supplementary Table S5.** Multivariate Cox analysis of clinical risk factors

| Feature | P-value | HR^*^ | 95%CI^†^ |
| --- | --- | --- | --- |
| Age | 0.119 | 1.42 | 0.91-2.20 |
| Gender (Male) | 0.147 | 2.05 | 0.78-5.40 |
| N-stage | 0.011 | 1.94 | 1.16-3.22 |
| M-stage | 0.046 | 4.10 | 1.03-16.33 |
| * HR, hazard ratio | | | |
| ^†^ CI, confidence interval | | | |

**Supplementary Table S6.** Multivariate Cox regression analysis of

RAD score and clinical factors

| Feature | P-value | HR^*^ | 95%CI^†^ |
| --- | --- | --- | --- |
| Age | 0.004 | 1.07 | 1.02-1.13 |
| Gender (Male) | 0.011 | 3.85 | 1.37-10.86 |
| N-stage | 0.083 | 1.66 | 0.94-2.93 |
| M-stage | 0.003 | 10.07 | 2.17-46.84 |
| Histology | 0.206 | 1.81 | 0.72-4.55 |
| RAD score | 5.752E-06 | 9.97 | 3.69-26.95 |
| * HR, hazard ratio |  |  |  |
| ^†^ CI, confidence interval |  |  |  |

**Supplementary Table S7.** Results of the t-test comparing gene expression between high and low PET radiomic (RAD) risk cohorts, and Cox univariate survival analysis.

|  | Differential expression  between risk groups | | | Cox univariate  analysis | |  |
| --- | --- | --- | --- | --- | --- | --- |
| Gene | **Statistic** | **P-value** | **FDR** | **P-value** | **CI*** | **Pathway** |
| TP53 | 1.72 | 9.29E-02 | 0.11 | 5.45E-02 | 0.67 | WP_GLYCOLYSIS_IN_SENESCENCE |
| EGFR | 2.06 | 4.80E-02 | 0.14 | 3.32E-01 | 0.66 | REACTOME_EGFR_TRANSACTIVATION_BY_GASTRIN |
| ZC3HC1 | 3.65 | 1.21E-03 | 0.06 | 3.57E-02 | 0.66 | REACTOME_SIGNALING_BY_ALK_IN_CANCER |
| HIF1A | 3.19 | 2.78E-03 | 0.02 | 2.72E-01 | 0.56 | WP_HIF1A_AND_PPARG_REGULATION_OF_GLYCOLYSIS |
| LDHA | 2.48 | 1.74E-02 | 0.03 | 5.24E-01 | 0.61 | WP_AEROBIC_GLYCOLYSIS |
| LDHB | 2.53 | 1.59E-02 | 0.06 | 9.24E-02 | 0.66 | WP_GLYCOLYSIS_AND_GLUCONEOGENESIS |
| HK1 | 2.80 | 7.96E-03 | 0.02 | 3.75E-01 | 0.64 | WP_AEROBIC_GLYCOLYSIS |
| GPI | 3.43 | 1.46E-03 | 0.01 | 6.68E-02 | 0.67 | REACTOME_GLUCONEOGENESIS |
| ALDOA | 4.31 | 1.36E-04 | 0.00 | 1.44E-02 | 0.71 | WP_AEROBIC_GLYCOLYSIS |
| GAPDH | 1.86 | 7.15E-02 | 0.09 | 5.50E-01 | 0.65 | WP_AEROBIC_GLYCOLYSIS |
| ENO1 | 2.55 | 1.50E-02 | 0.03 | 6.16E-01 | 0.56 | WP_AEROBIC_GLYCOLYSIS |
| PKM | 1.88 | 7.11E-02 | 0.09 | 1.26E-01 | 0.65 | WP_AEROBIC_GLYCOLYSIS |
| PRPS1 | 3.03 | 4.87E-03 | 0.02 | 4.25E-01 | 0.61 | WP_PURINE_METABOLISM |
| PAICS | 3.69 | 6.91E-04 | 0.03 | 1.12E-02 | 0.77 | KEGG_PURINE_METABOLISM |
| IMPDH1 | 3.78 | 5.34E-04 | 0.00 | 5.69E-02 | 0.58 | WP_PURINE_METABOLISM |
| ADSL | 2.94 | 5.95E-03 | 0.02 | 5.92E-01 | 0.44 | WP_PURINE_METABOLISM |
| PDHA1 | 2.45 | 2.00E-02 | 0.06 | 2.84E-01 | 0.66 | WP_GLYCOLYSIS_AND_GLUCONEOGENESIS |
| SLC2A1 | 2.39 | 2.25E-02 | 0.03 | 1.38E-01 | 0.60 | WP_AEROBIC_GLYCOLYSIS |
| G6PD | 2.90 | 6.25E-03 | 0.04 | 2.07E-01 | 0.68 | WP_GLYCOLYSIS_IN_SENESCENCE |
| COX5A | 2.54 | 1.57E-02 | 0.10 | 3.83E-01 | 0.63 | REACTOME_TP53_REGULATES_METABOLIC_GENES |
| CYCS | 4.04 | 3.15E-04 | 0.01 | 1.52E-01 | 0.69 | KEGG_P53_SIGNALING_PATHWAY |
| RAD51 | 2.75 | 1.11E-02 | 0.11 | 6.72E-02 | 0.70 | HALLMARK_DNA_REPAIR |
| ERCC2 | 3.34 | 2.05E-03 | 0.06 | 2.16E-02 | 0.63 | HALLMARK_DNA_REPAIR |
| CUL4B | 2.97 | 5.77E-03 | 0.14 | 1.28E-01 | 0.66 | REACTOME_DNA_REPAIR |
| PSMD12 | 4.08 | 2.20E-04 | 0.00 | 1.43E-02 | 0.71 | REACTOME_G1_S_DNA_DAMAGE_CHECKPOINTS |
| CHEK1 | 2.38 | 3.02E-02 | 0.08 | 8.83E-03 | 0.74 | REACTOME_CHK1_CHK2_CDS1_CDK1 |
| AKT1S1 | 3.07 | 6.28E-03 | 0.07 | 8.37E-02 | 0.61 | WP_P53_TRANSCRIPTIONAL_GENE_NETWORK |
| CCNE1 | 2.31 | 3.18E-02 | 011 | 1.66E-01 | 0.63 | REACTOME_G1_S_DNA_DAMAGE_CHECKPOINTS |
| CCNB1 | 2.19 | 4.19E-02 | 0.07 | 1.58E-02 | 0.68 | REACTOME_INACTIVATION_OF_CYCLIN_B_CDK1_COMPLEX |
| PLK1 | 3.91 | 6.45E-04 | 0.02 | 3.83E-05 | 0.83 | REACTOME_MITOTIC_G2_G2_M_PHASES |
| DUSP14 | 3.41 | 1.54E-03 | 0.08 | 1.24E-01 | 0.63 | KEGG_MAPK_SIGNALING_PATHWAY |
| DUSP7 | 4.00 | 2.72E-04 | 0.05 | 7.26E-04 | 0.67 | WP_MAPK_SIGNALING_PATHWAY |
| BIRC5 | 2.84 | 9.14E-03 | 0.07 | 2.05E-01 | 0.63 | REACTOME_SUMOYLATION_OF_DNA_REPLICATION_PROTEINS |
| PCK2 | 3.49 | 1.26E-03 | 0.01 | 2.22E-01 | 0.66 | REACTOME_GLUCONEOGENESIS |
| VDAC1 | 2.19 | 3.57E-02 | 0.11 | 2.52E-03 | 0.73 | REACTOME_PYRUVATE_METABOLISM |
| PGK2 | 1.70 | 9.84E-02 | 0.18 | 4.26E-02 | 0.61 | WP_GLYCOLYSIS_AND_GLUCONEOGENESIS |
| CD44 | 2.03 | 5.15E-02 | 0.22 | 2.21E-02 | 0.65 | HALLMARK_ GLYCOLYSIS |
| * CI, Concordance Index | | |  |  |  |  |

**Supplementary Table S8.** Genes associated with tumorigenesis activity, where more than 70% of patients with gene gain or loss belong to the high PET radiomic (RAD) risk cohort.

| Gene | CNV gain/loss | % of patients with CNV gain/loss  among all patients | % of patients with CNV gain/loss  belong high risk cohort | % of patients with CNV gain/loss  belong low risk cohort |
| --- | --- | --- | --- | --- |
| TP53 | loss | 50.00 | 80.00 | 20.00 |
| ERCC1 | loss | 25.00 | 80.00 | 20.00 |
| ERCC2 | loss | 25.00 | 80.00 | 20.00 |
| BRCA1 | loss | 20.00 | 87.50 | 12.50 |
| BRCA2 | loos | 52.50 | 80.95 | 19.05 |
| PTEN | loss | 40.00 | 75.00 | 25.00 |
| EGFR | gain | 62.50 | 84.00 | 16.00 |
| HIF1A | gain | 17.50 | 75.00 | 25.00 |
| PDHA1 | gain | 25.00 | 80.00 | 20.00 |
| G6PD | gain | 27.50 | 81.82 | 18.18 |
| RAD51 | gain | 25.00 | 80.00 | 20.00 |
| SLC2A1 | gain | 32.50 | 84.62 | 15.38 |
| CUL4B | gain | 25.00 | 70.00 | 30.00 |
| CCNE1 | gain | 40.00 | 68.75 | 31.25 |
| PLK1 | gain | 22.50 | 77.78 | 22.22 |
| BIRC5 | gain | 42.50 | 82.35 | 17.65 |
| CD44 | gain | 20.00 | 75.00 | 25.00 |

**Supplementary Table S9.** Results of the t-test comparing gene expression between high and low semiquantitative (SQ) PET risk cohorts, and Cox univariate survival analysis.

|  | Differential expression  between risk groups | | | Cox univariate  analysis | |  |
| --- | --- | --- | --- | --- | --- | --- |
| Gene | **Statistic** | **P-value** | **FDR** | **P-value** | **CI*** | **Pathway** |
| EGFR | 2.17 | 3.75E-02 | 0.11 | 3.32E-01 | 0.66 | REACTOME_EGFR_TRANSACTIVATION_BY_GASTRIN |
| HIF1A | 2.77 | 9.69E-03 | 0.03 | 2.72E-01 | 0.56 | WP_HIF1A_AND_PPARG_REGULATION_OF_GLYCOLYSIS |
| LDHA | 1.93 | 6.68E-02 | 0.12 | 5.24E-01 | 0.61 | WP_GLYCOLYSIS_IN_SENESCENCE |
| HK1 | 2.69 | 9.69E-03 | 0.03 | 3.75E-01 | 0.64 | WP_AEROBIC_GLYCOLYSIS |
| HK2 | 2.57 | 1.92E-02 | 0.08 | 9.39E-01 | 0.47 | WP_GLYCOLYSIS_IN_SENESCENCE |
| GPI | 1.90 | 7.85E-02 | 0.13 | 6.68E-02 | 0.67 | WP_AEROBIC_GLYCOLYSIS |
| ALDOA | 1.45 | 1.66E-01 | 0.20 | 1.44E-02 | 0.71 | WP_AEROBIC_GLYCOLYSIS |
| ALDOC | 3.44 | 1.66E-03 | 0.01 | 4.21E-01 | 0.57 | WP_GLYCOLYSIS_IN_SENESCENCE |
| GAPDH | 1.74 | 1.03E-01 | 0.14 | 5.50E-01 | 0.65 | WP_AEROBIC_GLYCOLYSIS |
| ENO1 | 2.19 | 3.59E-02 | 0.08 | 6.16E-01 | 0.56 | WP_GLYCOLYSIS_IN_SENESCENCE |
| PKM | 2.91 | 7.84E-03 | 0.03 | 1.26E-01 | 0.65 | WP_GLYCOLYSIS_IN_SENESCENCE |
| PRPS1 | 3.04 | 5.10E-03 | 0.03 | 4.25E-01 | 0.61 | WP_PURINE_METABOLISM |
| ADSL | 4.58 | 2.07E-05 | 0.00 | 5.92E-01 | 0.44 | WP_PURINE_METABOLISM |
| GMPS | 3.39 | 2.38E-03 | 0.04 | 5.08E-01 | 0.62 | KEGG_PURINE_METABOLISM |
| SLC2A1 | 4.65 | 4.19E-05 | 0.00 | 1.38E-01 | 0.60 | WP_AEROBIC_GLYCOLYSIS |
| BRCA1 | 3.01 | 7.40E-03 | 0.03 | 2.35E-02 | 0.71 | REACTOME_G2_M_CHECKPOINTS |
| BRCA2 | 1.86 | 7.70E-02 | 0.17 | 3.93E-01 | 0.64 | HALLMARK_G2M_CHECKPOINT |
| ERCC1 | 2.18 | 4.06E-02 | 0.14 | 8.59E-01 | 0.50 | REACTOME_DNA_DOUBLE_STRAND_BREAK_REPAIR |
| COX5A | 3.73 | 8.17E-04 | 0.02 | 3.83E-01 | 0.63 | REACTOME_TP53_REGULATES_METABOLIC_GENES |
| CYCS | 2.63 | 1.62E-02 | 0.07 | 1.52E-01 | 0.69 | WP_DNA_DAMAGE_RESPONSE |
| RAD51 | 4.09 | 5.75E-04 | 0.01 | 6.72E-02 | 0.70 | WP_DNA_DAMAGE_RESPONSE |
| CUL4B | 2.65 | 1.88E-02 | 0.11 | 1.28E-01 | 0.66 | REACTOME_DNA_REPAIR |
| CHEK1 | 2.58 | 2.40E-02 | 0.06 | 8.83E-03 | 0.74 | REACTOME_CHK1_CHK2_CDS1_CDK1 |
| CHEK2 | 5.67 | 2.40E-02 | 0.00 | 5.68E-01 | 0.46 | REACTOME_CHK1_CHK2_CDS1_CDK1 |
| CCNB1 | 2.47 | 2.84E-02 | 0.04 | 1.58E-02 | 0.68 | REACTOME_G2_M_DNA_REPLICATION_CHECKPOINT |
| CCNB2 | 4.95 | 1.61E-04 | 0.00 | 2.10E-02 | 0.71 | REACTOME_G2_M_DNA_REPLICATION_CHECKPOINT |
| PLK1 | 2.19 | 4.61E-02 | 0.11 | 3.83E-05 | 0.83 | HALLMARK_G2M_CHECKPOINT |
| BIRC5 | 4.19 | 3.62E-04 | 0.00 | 2.05E-01 | 0.63 | REACTOME_CELL_CYCLE_CHECKPOINTS |
| PCK2 | 2.44 | 1.98E-02 | 0.09 | 2.22E-01 | 0.66 | REACTOME_GLUCONEOGENESIS |
| PGK1 | 3.91 | 4.10E-04 | 0.00 | 4.26E-02 | 0.61 | WP_AEROBIC_GLYCOLYSIS |
| PIK3CA | 2.65 | 1.27E-02 | 0.05 | 7.63E-01 | 0.58 | BIOCARTA_HER2 |
| PDK1 | 2.43 | 2.02E-02 | 0.17 | 1.68E-01 | 0.57 | HALLMARK_HYPOXIA |
| RRM1 | 5.14 | 7.99E-06 | 0.00 | 5.89E-02 | 0.71 | KEGG_PYRIMIDINE_METABOLISM |
| MKI67 | 2.64 | 1.94E-02 | 0.06 | 1.94E-02 | 0.64 | HALLMARK_G2M_CHECKPOINT |
| SERPINB5 | 2.51 | 1.96E-02 | 0.11 | 7.06E-01 | 0.59 | KEGG_P53_SIGNALING_PATHWAY |
| EZH2 | 3.50 | 1.28E-03 | 0.01 | 6.03E-01 | 0.60 | HALLMARK_G2M_CHECKPOINT |
| XRCC6 | 5.05 | 1.07E-05 | 0.00 | 4.35E-01 | 0.67 | REACTOME_DNA_DOUBLE_STRAND_BREAK_REPAIR |
| CD28 | -1.86 | 9.44E-02 | 0.20 | 5.36E-02 | 0.76 | KEGG_ALLOGRAFT_REJECTION |
| CD40LG | -2.64 | 3.24E-02 | 0.16 | 6.37E-01 | 0.67 | KEGG_ALLOGRAFT_REJECTION |
| IL2 | -1.78 | 1.15E-01 | 0.20 | 6.37E-01 | 0.60 | KEGG_ALLOGRAFT_REJECTION |
| CDKN2A | 1.64 | 1.17E-01 | 0.22 | 8.52E-01 | 0.62 | WP_G1_TO_S_CELL_CYCLE_CONTROL |
| * CI, Concordance Index | | |  |  |  |  |

**Supplementary Table S10.** Genes associated with tumorigenesis activity, where more than 70% of patients with gene gain or loss belong to the high semiquantitative (SQ) PET risk cohort.

| Gene | CNV gain/loss | % of patients with CNV gain/loss  among all patients | % of patients with CNV gain/loss  belong high risk cohort | % of patients with CNV gain/loss  belong low risk cohort |
| --- | --- | --- | --- | --- |
| CDKN2A | loss | 52.50 | 76.19 | 23.81 |
| ERCC1 | loss | 25.00 | 90.00 | 10.00 |
| ERCC2 | loss | 25.00 | 90.00 | 10.00 |
| BRCA1 | loss | 20.00 | 100.00 | 0.00 |
| BRCA2 | loss | 52.50 | 85.71 | 14.29 |
| PTEN | loss | 40.00 | 75.00 | 25.00 |
| EGFR | gain | 62.50 | 88.00 | 12.00 |
| HIF1A | gain | 17.50 | 85.71 | 14.29 |
| RAD51 | gain | 25.00 | 100.00 | 0.00 |
| CHEK2 | loss | 17.50 | 85.71 | 14.29 |
| SLC2A1 | gain | 32.50 | 92.31 | 7.69 |
| CUL4B | gain | 25.00 | 100.00 | 0.00 |
| BIRC5 | gain | 42.50 | 76.47 | 23.53 |
| HK2 | gain | 40.00 | 81.25 | 18.75 |
| CCNB2 | gain | 25.00 | 100.00 | 0.00 |
| EZH2 | gain | 32.50 | 92.31 | 7.69 |

**Supplementary Table S11.** The range and distribution of radiomic features and risk scores across the samples in Data_1.

|  | Normalized values | | | | | Calculated values | | | | | | | | | |
| --- | --- | --- | --- | --- | --- | --- | --- | --- | --- | --- | --- | --- | --- | --- | --- |
| Feature | Min | Q1 | Median | Q3 | Max | Min | | Q1 | | Median | | Q3 | | Max | |
| GLNU | -0.74 | -0.59 | -0.38 | 0.11 | 4.01 | 2.67 | | 7.25 | | 13.70 | | 28.73 | | 147.38 | |
| SALGLE | -0.74 | -0.57 | -0.35 | 0.26 | 5.62 | 0.0002 | | 0.006 | | 0.01 | | 0.03 | | 0.20 | |
| IV | -4.43 | -0.59 | 0.15 | 0.70 | 2.30 | 0.28 | | 0.42 | | 0.45 | | 0.47 | | 0.53 | |
| BN | -0.45 | -0.40 | -0.29 | -0.12 | 7.82 | 0.21 | | 1.09 | | 2.96 | | 6.12 | | 149.23 | |
| MCC | -2.15 | -0.80 | 0.04 | 0.61 | 3.31 | 0.10 | | 0.26 | | 0.36 | | 0.43 | | 0.75 | |
| Risk score | **Risk values** | | | | |  | |  | |  | |  | |  | |
| *RAD*^*^ *Score* | -5.24 | -0.40 | 0.22 | 0.99 | 3.44 |  | |  | |  | |  | |  | |
| *SQ*^†^ *Score* | -0.21 | -0.14 | -0.04 | 0.11 | 0.84 |  | |  | |  | |  | |  | |
| *CLIN*^‡^ *Score* | -0.89 | 0.46 | 0.77 | 1.12 | 2.09 |  | |  | |  | |  | |  | |
| *NOM*^§^ *Score* | 4.27 | 6.66 | 7.48 | 8.71 | 10.83 |  | |  | |  | |  | |  | |
| * RAD: Radiomic model | | | | | | |  | |  | |  | |  | |  |
| ^†^  SQ: Semiquantitative model | | | | | | | | | | | | | | | |
| ^‡^ CLIN: Clinical model | | | | | | | | | | | | | | | |
| ^§^ NOM: Nomogram | | | | | | | | | | | | | | | |

## Supplementary Figures





**Supplementary Figure S1.** Funnel graph showing subject exclusion. Step 1: excluded cases with missing or poor-quality PET scans. Step 2: excluded cases lacking clinical or survival data. Final cohort includes subjects meeting both criteria.


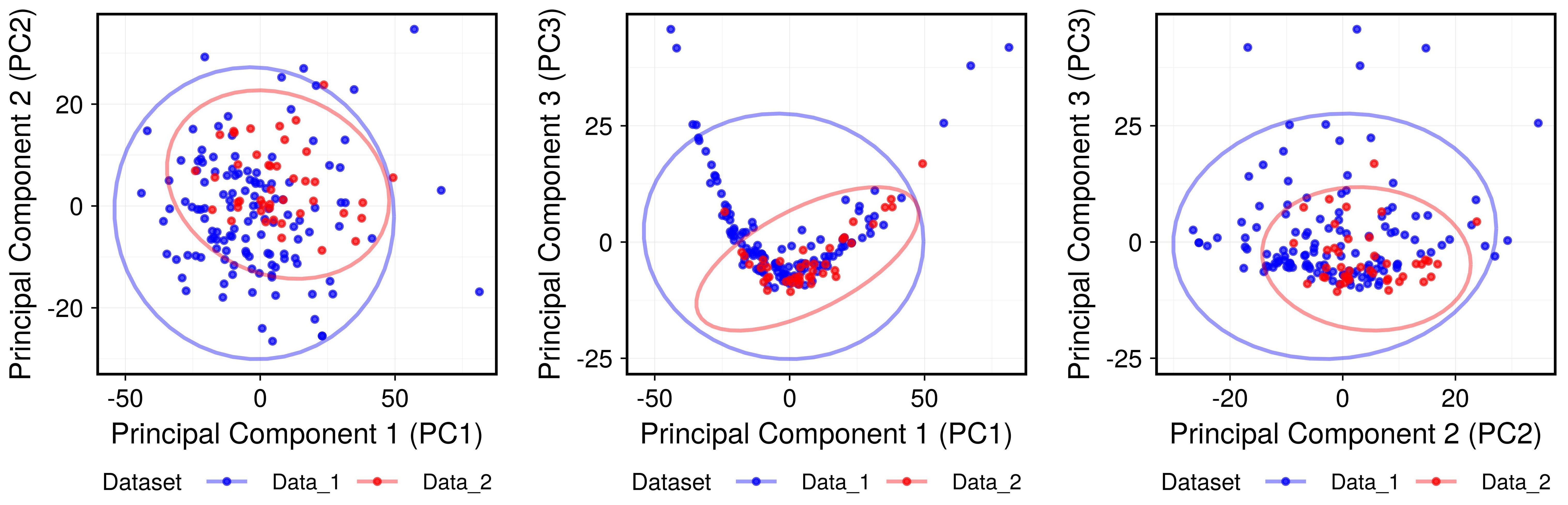


**Supplementary Figure S2.** Representation of the first three components from principal component analysis (PCA) of PET radiomic features, used to assess congruency of Data1 and Data2. The difference between the datasets was tested using the MANOVA test. Although the p-value was significant (p < 0.05), the partial eta-squared value was 0.04, which is generally considered a small effect. This is especially relevant here, as the PCA plots visually demonstrate consistent overlaps between the datasets.


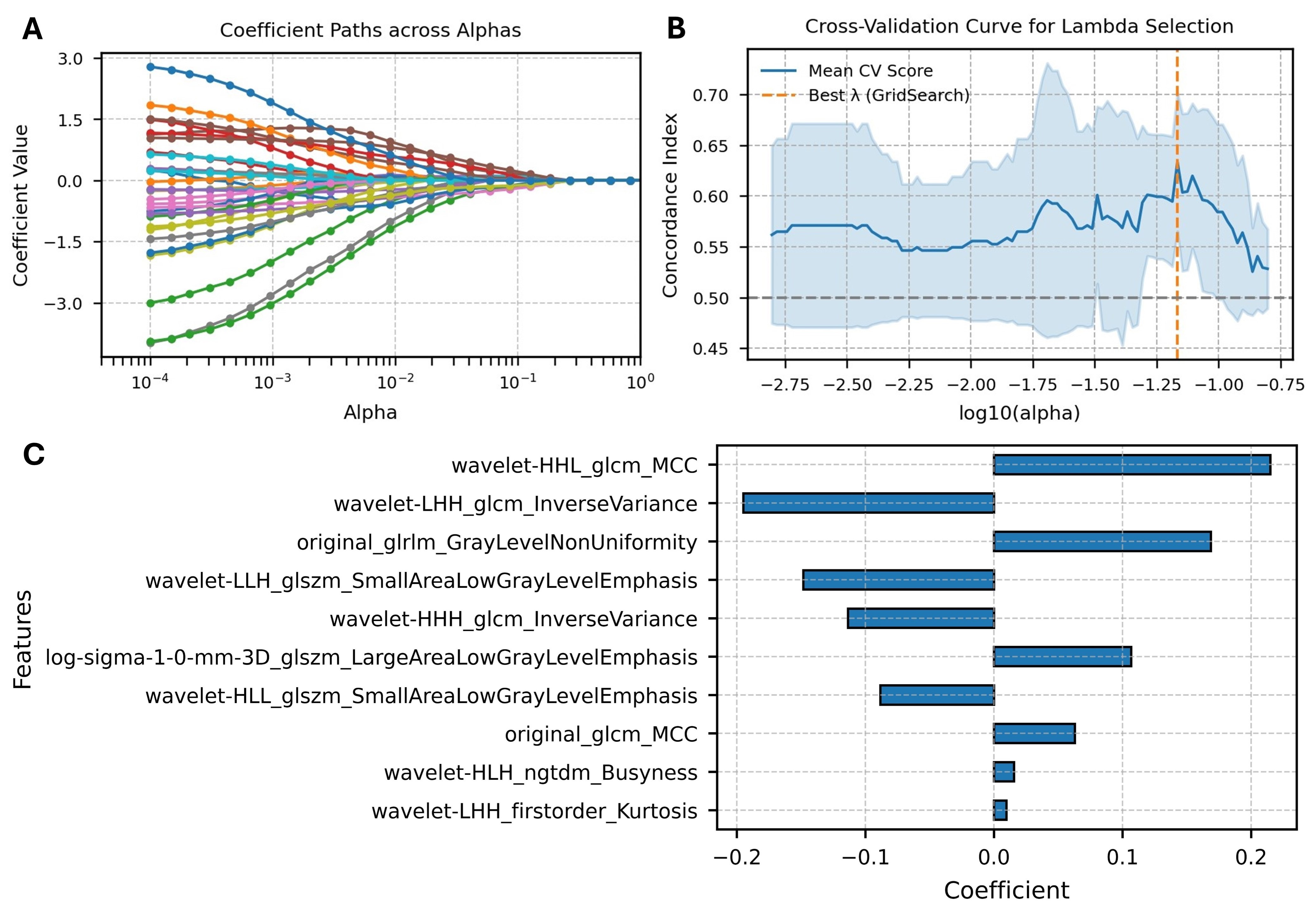


**Supplementary Figure S3.** (A) Regularization paths of ffeature coefficients across a sequence of λ (lambda) values using Coxnet with LASSO (l1_ratio=0.9). (B) Cross-validation results for selecting the optimal λ in Coxnet LASSO. The mean concordance index (C-index) with standard deviation is plotted against λ on a logarithmic scale. The vertical dashed line indicates the λ value that yielded the highest average performance. (C) Feature coefficients from the best-performing Coxnet LASSO model selected via cross-validation.


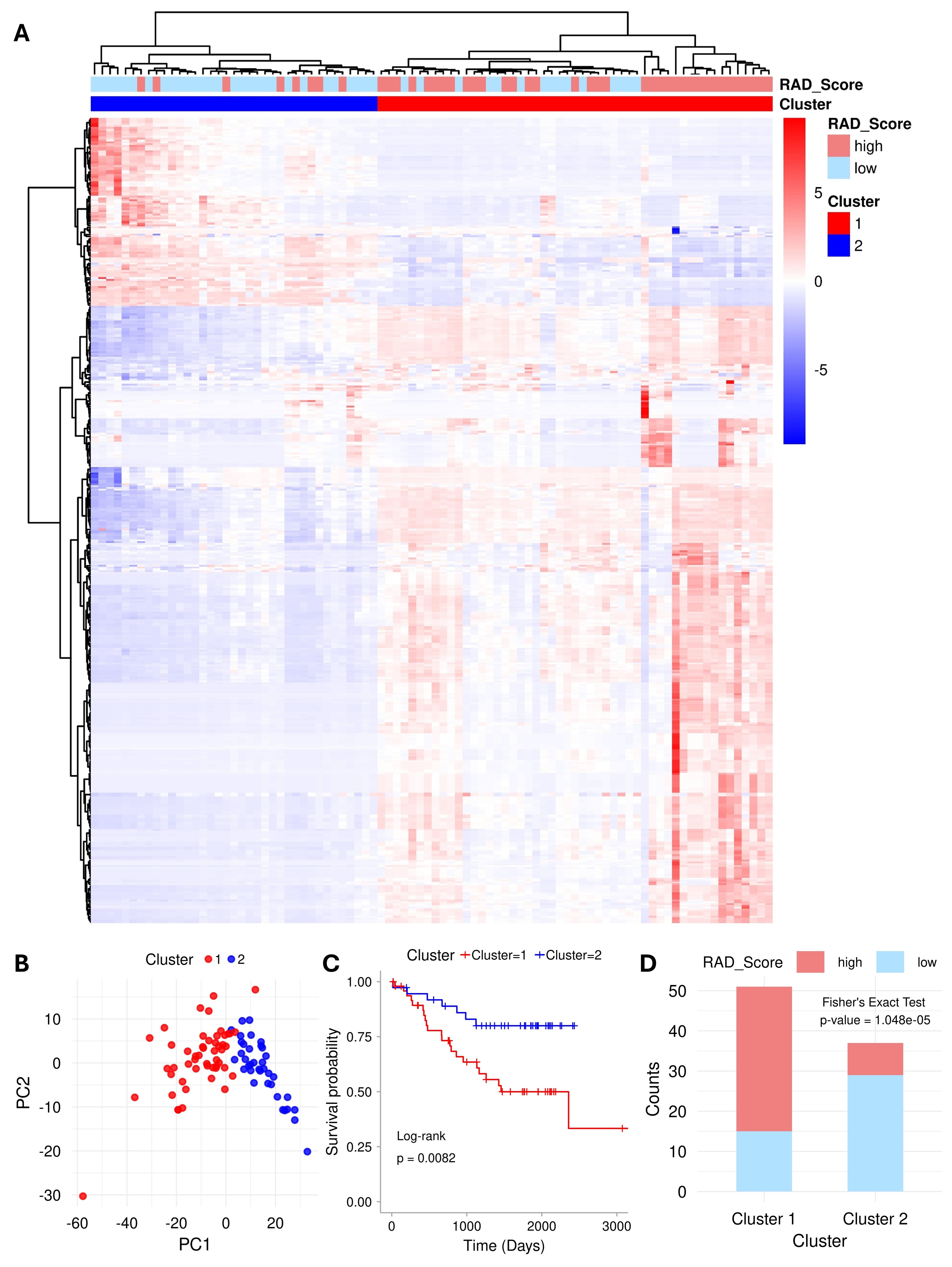


**Supplementary Figure S4.** Clustering-based analysis of radiomic features. (A) Heatmap showing the unsupervised clustering of patients (columns) based on 445 radiomic features (rows), revealing two distinct clusters. (B) PCA plot illustrates clear separation between the two clusters in component space. (C) KM survival curves demonstrating significantly different overall survival between the identified clusters (log-rank test, *p* < 0.01). (D) Association between RAD-score–based risk groups and clustering results, assessed using Fisher’s Exact Test (*p* = 0.00001).


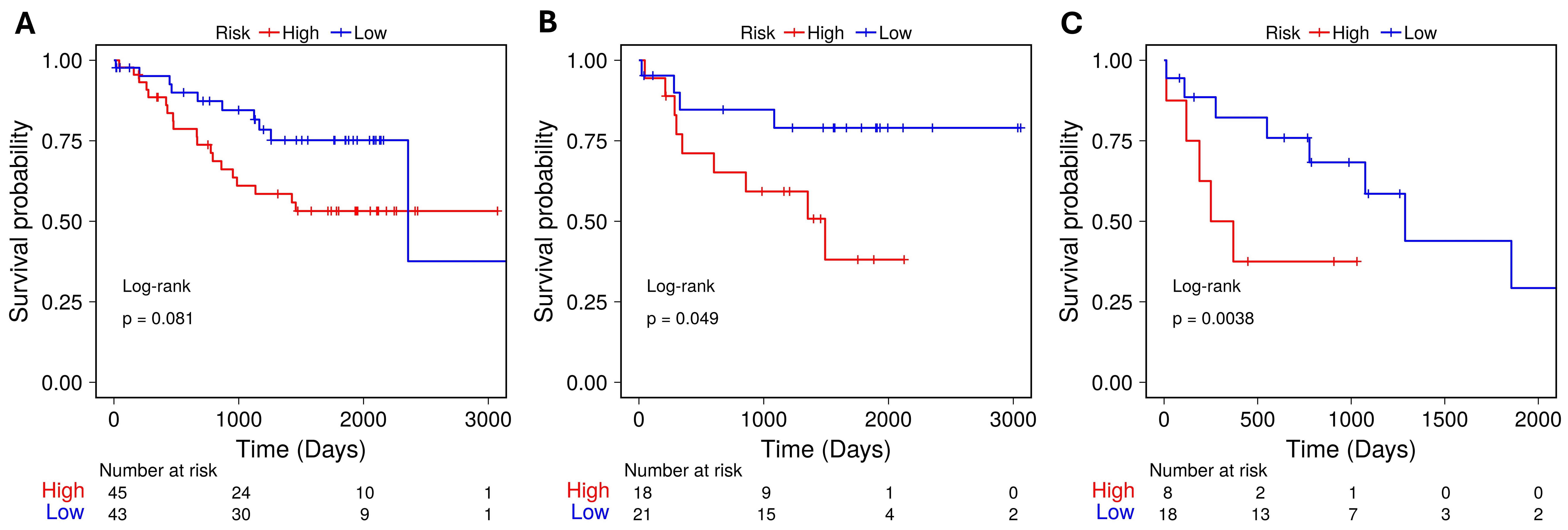


**Supplementary Figure S5.** Kaplan-Meier (KM) plots of the clinical model in: (A) train, (B) validation, and (C) test data.


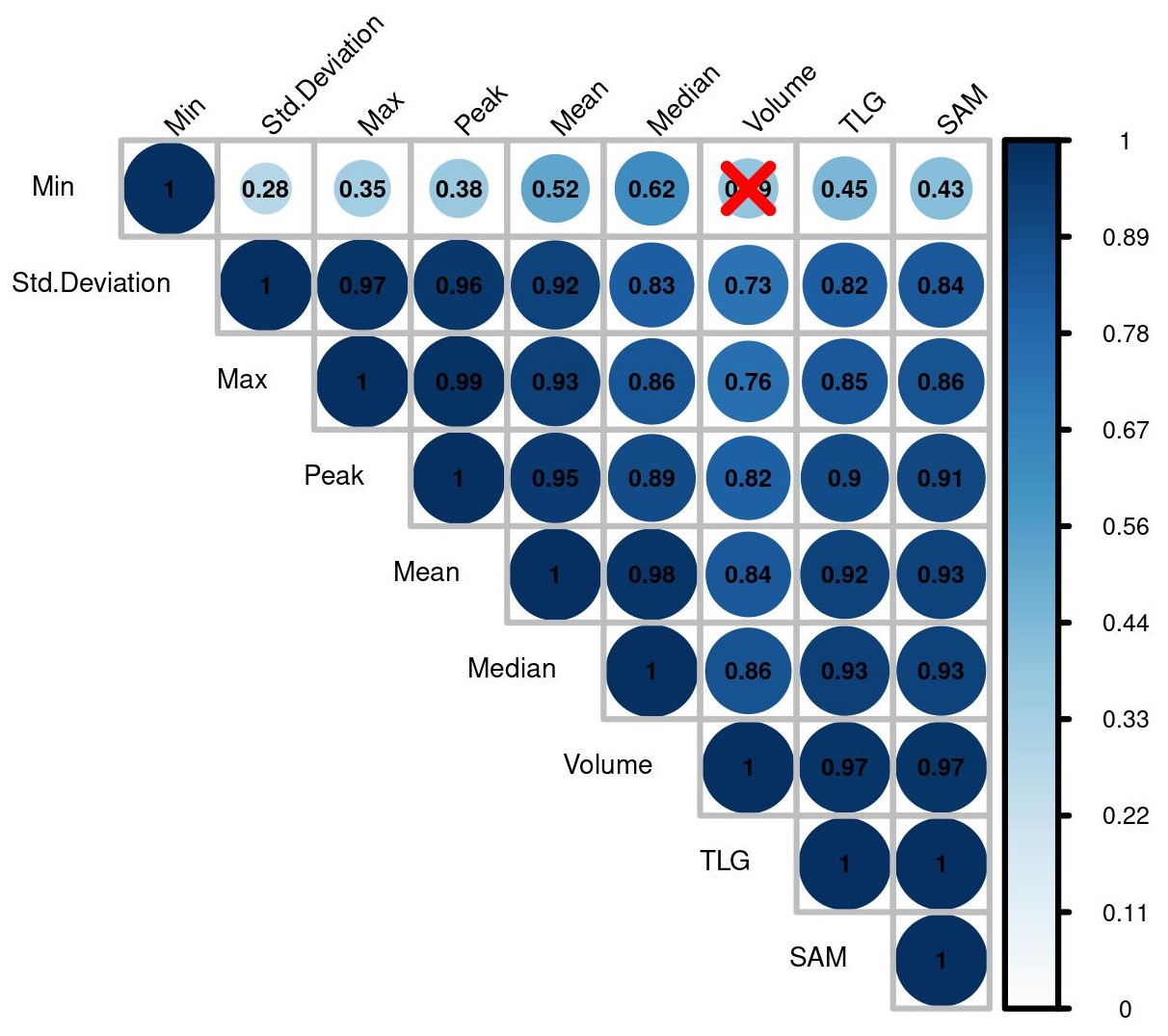


**Supplementary Figure S6.** Pearson correlation assessment of semiquantitative (SQ) PET features. The red cross mark indicates insignificant correlations.


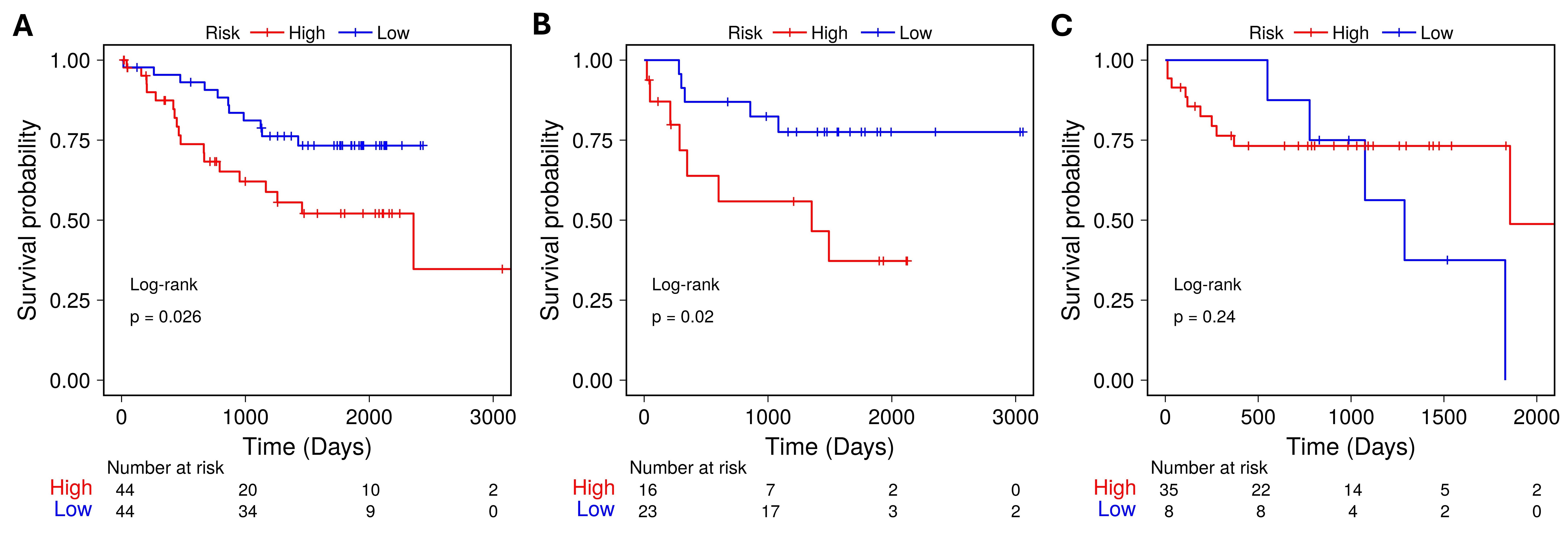


**Supplementary Figure 7.** Kaplan-Meier (KM) plots of the PET semiquantitative model in: (A) train, (B) validation, and (C) test data. The conventional model included one feature, SUVmax (P-value: 0.160; HR: 1.22; 95%CI: 0.92-1.62).


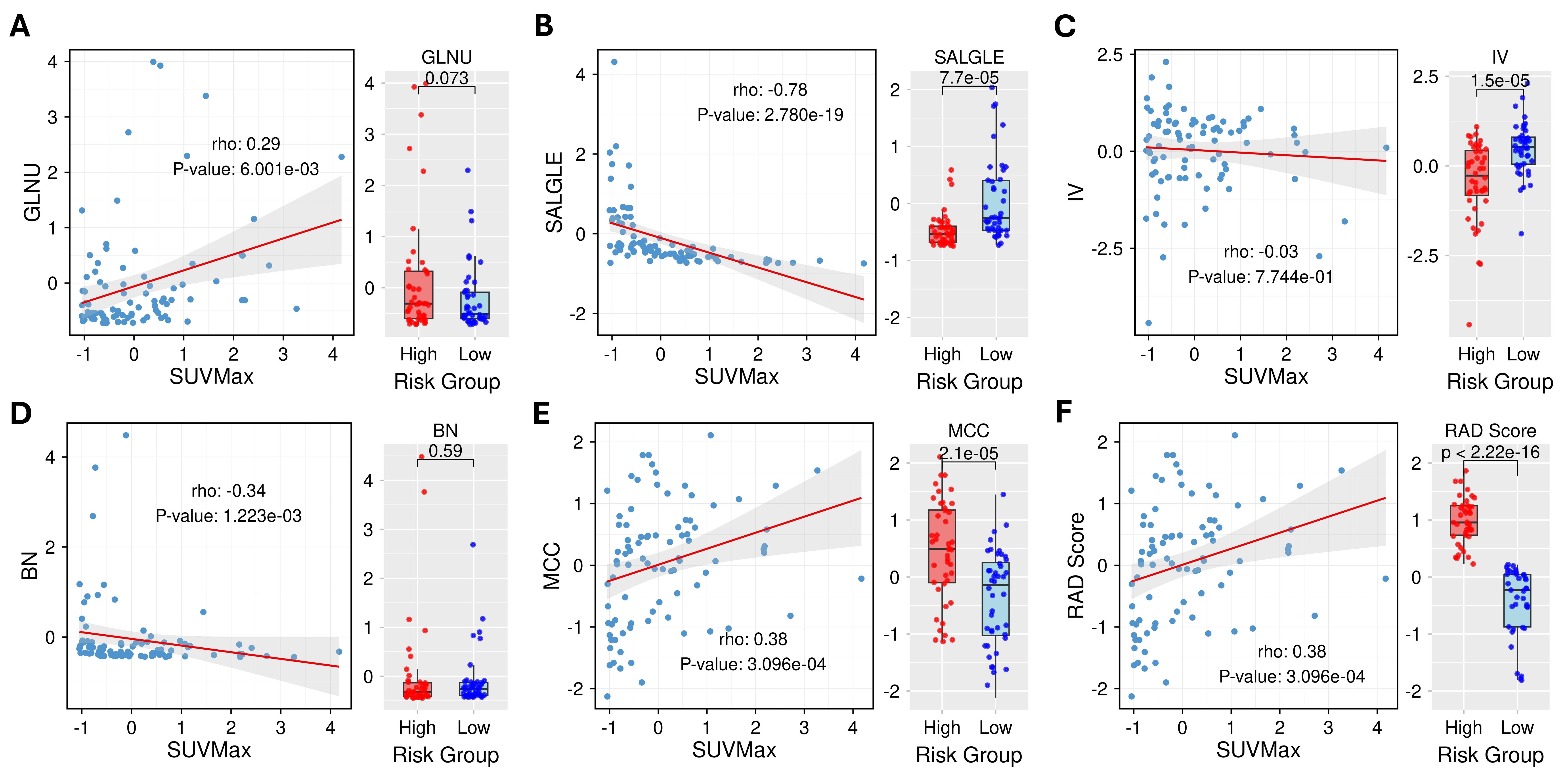


**Supplementary Figure S8.** Correlation and difference assessments between maximum standardized uptake value (SUV_max_) or SQ score (
$\text{SQ score}= (\text{SUVmax) }\times\left( 0.20 \right)$) and (A-E) five radiomic features included in the PET radiomic model, as well as (F) the radiomic risk (RAD) score. Difference assessments include comparisons of RAD score and radiomic features between high- and low-risk groups based on the semiquantitative (SQ) score. Radiomic features: *original_glrlm_GrayLevelNonUniformity* (GLNU), *SmallAreaLowGrayLevelEmpasis* (SALGLE), *Wavelet_LHH_glcm_InverseVariance* (IV), *wavelet-HLH_ngtdm_Busyness* (BN), and *wavelet-HHL_glcm_MCC* (MCC).


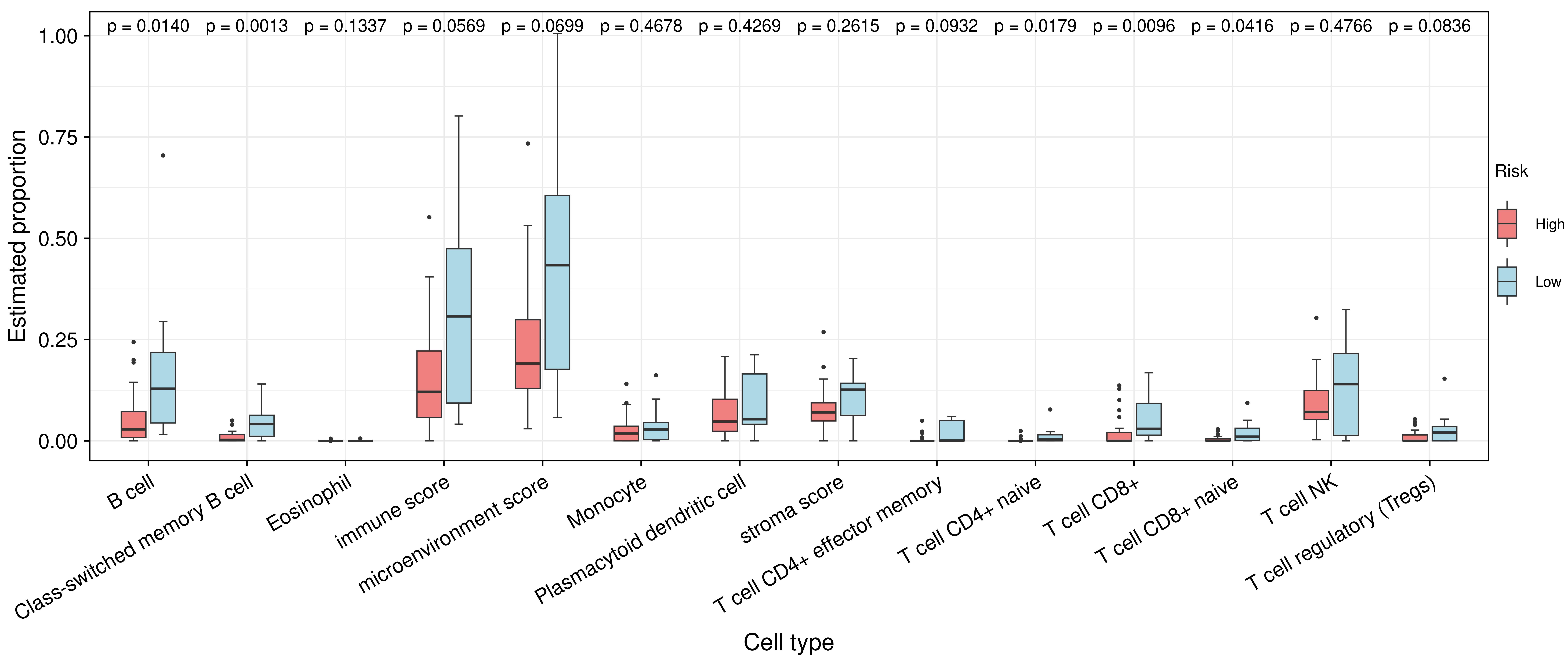


**Supplementary Figure S9.** Inferred immune cell infiltration levels within the tumor microenvironment across high- and low-risk cohorts as derived from *xCell* in R. Statistical significance was assessed using Wilcoxon signed-rank test.


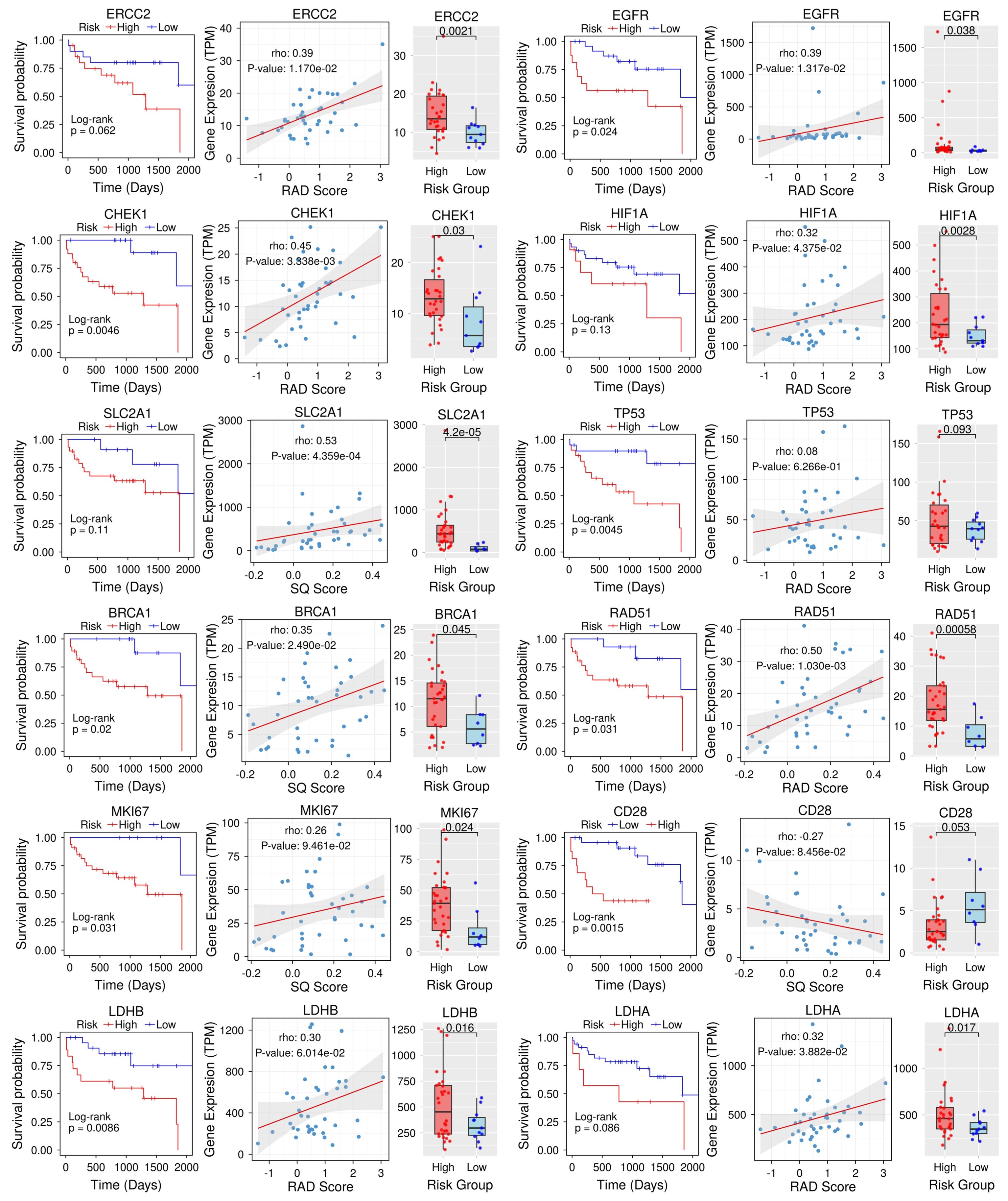


**Supplementary Figure S10.** Integrated analysis of genes associated with PET radiomic (RAD) and PET semiquantitative (SQ) risk scores in the external validation cohort (TCGA, Data_2). For each gene, (i) correlation analysis was performed to assess the association between gene expression levels and RAD/SQ scores, (ii) differential expression was evaluated between high- and low-risk cohorts (defined by RAD and SQ score thresholds) using the Wilcoxon rank-sum test, and (iii) prognostic significance was determined via Kaplan-Meier (KM) survival analysis stratified by gene expression levels. Genes shown are those significantly associated with at least one metric.
